# How stakeholder requirements constrain drone-based medical delivery: probabilistic sizing of eVTOL aircraft at high altitude

**DOI:** 10.1038/s41598-026-54743-2

**Published:** 2026-06-10

**Authors:** Victor Alulema, Gloria Acosta-Vargas, Cesar Guevara, Patricia Acosta-Vargas

**Affiliations:** 1 Department of Mechanical Engineering, Laboratory of Unmanned Aerial Systems (LUAS-EPN), ATA Research Group, Escuela Politécnica Nacional, Quito, 170143 Ecuador; 2 EAVISE, PSI, Department of Electrical Engineering, KU Leuven, Jan Pieter de Nayerlaan 5, Sint-Katelijne-Waver, Belgium; 3 Medicine Faculty, Pontificia Universidad Católica del Ecuador, Quito, 170143 Ecuador; 4 Quantitative Methods Department, CUNEF University, Madrid, 28040 Spain; 5 Intelligent and Interactive Systems Laboratory, Ingeniería Industrial, FICA, Universidad de Las Américas, Quito, 170125 Ecuador

**Keywords:** EVTOL sizing, Uncertainty quantification, Probabilistic design, Sobol sensitivity analysis, Medical delivery UAV, Requirement analysis, Engineering, Mathematics and computing

## Abstract

Drone-based medical delivery can overcome geographic barriers to healthcare in highland regions, but designing aircraft for this mission requires navigating uncertainty that originates outside engineering: physicians define cold-chain requirements, regulators mandate safety systems, and health planners set delivery speed targets, each imposing design penalties whose magnitudes are uncertain. No existing aircraft sizing framework propagates this requirement-origin uncertainty alongside classical technological uncertainty. Here we introduce Requirement Impact Kits (RIKs)—composable modules with uncertain engineering coefficients—within a three-level probabilistic framework that distinguishes requirement-scenario, engineering-translation, and parameter-level uncertainty. Applied to eVTOL medical delivery at 2,800 m altitude in the Quito Metropolitan District, comparing Lift+Cruise, Tilt-Rotor, and Multirotor architectures, the framework reveals that stakeholder decisions dominate the design space: demanding urgent delivery reduces aircraft feasibility by 24 percentage points—four times the impact of adding cold-chain thermal management. Sobol sensitivity decomposition shows that 98% of parameter-level variance is epistemic and reducible through rotor testing, while battery energy density contributes less than 2%. The Lift+Cruise architecture achieves the highest robustness, and an altitude–cruise speed feasibility map provides health system planners with a quantitative tool for negotiating delivery time requirements against operational reliability.

## Introduction

Timely access to blood products and emergency medical supplies is a critical determinant of patient outcomes, yet in many regions this access is constrained by geography, infrastructure, and climate. Approximately two billion people worldwide live beyond reliable reach of essential medicines^1^, and the problem is most acute in highland developing regions—the Andes, the East African Rift, the Himalayas, and the Central Asian plateaus—where unpaved roads, seasonal landslides, and extreme topography can delay ground-based deliveries by hours or render them impossible altogether. Over 150 million people live above 2,500 m in cities such as Quito, La Paz, Bogotá, Addis Ababa, and Kathmandu, where health facilities are often dispersed across mountainous terrain connected by road networks vulnerable to congestion, weather, and natural hazards^2,3^. For time-critical supplies—blood for obstetric hemorrhage, antivenoms, vaccines requiring cold-chain integrity—even moderate delays can be fatal.

Unmanned Aerial Vehicles (UAVs, commonly called drones) have emerged as a transformative solution to this last-mile problem. In Rwanda, Zipline’s autonomous fixed-wing drones deliver over 35% of the national blood supply outside the capital, serving facilities within an 80 km radius from two distribution hubs; a retrospective analysis in *The Lancet Global Health* found that drone delivery reduced median delivery times by 71% and decreased blood product expiration rates^4,5^. Feasibility studies across India, Canada, and Lebanon have confirmed that blood product quality—hemolysis markers, coagulation function, and temperature stability—is preserved after UAV transport over distances of 13–30 km with payloads up to 1.9 kg^6,7^. In the Indian Himalayas, drones operating at approximately 3,400 m altitude reduced medicine delivery times from two hours by road to under ten minutes by air, demonstrating viability in extreme cold (−10°C) and high-altitude conditions^1^.

However, these successes mask a critical engineering gap. The platforms used in current medical drone programs fall into two categories: fixed-wing catapult-launch systems that require dedicated infrastructure and cannot operate from unprepared sites (Zipline), and small multirotors that can take off and land anywhere but are limited to ranges below approximately 20 km due to the absence of a wing for efficient cruise flight. Neither architecture class addresses the needs of health systems that require point-to-point delivery from hospital rooftops or clinic courtyards over medium ranges (20–60 km) across mountainous terrain. Electric Vertical Take-Off and Landing (eVTOL) aircraft—hybrid configurations that combine the vertical flight capability of multirotors with the cruise efficiency of fixed-wing platforms—could bridge this gap^8,9^. Yet designing an eVTOL specifically for medical delivery at high altitude introduces sizing challenges that go beyond simply scaling an existing platform. At 2,800 m MSL, the density ratio $\sigma _\rho = \rho /\rho _{SL}$ drops to approximately 0.74 under standard atmospheric conditions. This degrades performance through coupled mechanisms: hover power increases by 16% (scaling as $P \propto T^{3/2}/\sqrt{\rho }$), stall speed rises by 16% ($V_{stall} \propto 1/\sqrt{\sigma _\rho }$), and complex terrain necessitates climb profiles that can consume 30–40% of total mission energy^3^. Critically, motor efficiency maps, propeller performance curves, and battery discharge characteristics provided by component manufacturers are calibrated for sea-level conditions; extrapolating them to high altitude introduces epistemic uncertainty that compounds with the operational variability of the environment^10^.

The deeper challenge, however, is not atmospheric but organizational. Medical delivery missions impose requirements that originate from non-engineering stakeholders—physicians, regulators, health logistics planners—whose qualitative specifications must be translated into quantitative engineering parameters before they can enter the aircraft sizing loop. A physician specifies that blood products must be maintained at 1–6°C during transport; a thermal engineer translates this into a cooling system with uncertain mass ($\Delta W = a + b \cdot W_{pay}$, where *b ∈ [0.2, 0.45]* kg/kg depending on insulation quality and phase-change material selection) and a continuous power draw of 15–30 W. A regulator mandates safe flight termination after motor failure; a propulsion engineer translates this into a ballistic parachute system whose mass scales with aircraft weight through an uncertain fraction (4–8% of maximum take-off weight), creating a feedback loop where heavier aircraft need larger recovery systems. A health planner demands 30-minute delivery over 20 km; an aerodynamicist translates this into a cruise speed requirement that activates an energy constraint absent from the lower-speed regime. Each translation step introduces *requirement-origin uncertainty*—uncertainty that is neither captured by classical parameter-level UQ methods nor visible to the stakeholder who defined the requirement. Program managers currently lack a quantitative basis for evaluating whether relaxing a delivery speed requirement would yield greater design benefit than investing in better battery technology, because these two uncertainty sources are not represented within the same analytical framework.

This layered uncertainty structure has no precedent in the eVTOL sizing literature, which exhibits three specific gaps. First, the classical thrust-to-weight (*T*/*W*) versus wing loading (*W*/*S*) constraint diagram—the foundational tool of conceptual aircraft sizing^11,12^—has not been extended probabilistically for eVTOL aircraft. Computational frameworks such as NASA’s NDARC and the open-source SUAVE platform produce point designs from fixed input parameters^9,13^, and recent robust sizing methods for powered-lift eVTOL types use hybrid root-finding algorithms without uncertainty propagation^14^. The consequence is a persistent disconnect: designers establish feasible design points using deterministic constraints, then discover infeasibility when actual parameter variability is encountered during prototyping or flight testing—a risk that is amplified at high altitude, where the density-coupled performance degradation widens the gap between nominal and worst-case conditions. Second, uncertainty quantification studies in eVTOL design propagate technological uncertainties (battery energy density, aerodynamic coefficients) through Monte Carlo or Polynomial Chaos methods^15–17^ but treat operational requirements as deterministic inputs. This means that the relative impact of a stakeholder’s delivery speed demand versus an engineer’s battery technology assumption cannot be compared within the same framework—precisely the comparison that a program manager needs to make when negotiating requirements with health logistics planners. Third, Sobol-based global sensitivity analysis—the standard methodology for variance decomposition^18,19^ that has been recommended for conceptual aircraft design by DLR^20^ and applied to hybrid-electric powertrain sizing^21^—has not been applied to eVTOL vehicle sizing. Without Sobol decomposition, designers lack a principled tool for prioritizing which uncertain parameters to characterize first through testing, and which to accept as irreducible variability. Similarly, configuration comparison studies between Lift+Cruise (L+C), Tilt-Rotor (T-R), and Multirotor (MR) architectures^22–24^ remain deterministic, with no standardized robustness index for architecture selection under uncertainty. These three gaps reflect a broader disconnect between two bodies of literature that have developed largely independently: the medical drone operations community, which has demonstrated clinical feasibility but does not engage with aircraft sizing trade-offs^1,4,7^, and the aerospace conceptual design community, which has developed sophisticated sizing tools but does not represent application-specific requirements as uncertain inputs^13,14^. Bridging these two communities requires a framework that speaks both languages—expressing aircraft feasibility in terms that health planners can act on, while representing medical requirements in terms that sizing algorithms can propagate.

This paper addresses these gaps by formalizing the requirement-to-sizing interface as a first-class source of uncertainty. The proposed Requirement Impact Kits differ from three established methodological families. Quality Function Deployment (QFD) and requirement allocation methods^25^ translate stakeholder needs into engineering specifications but do so deterministically and without coupling to physics-based sizing—they produce fixed targets, not uncertain parameters within a constraint loop. Robust design methods (Taguchi, Reliability-Based Design Optimization^26^) propagate parameter uncertainty but treat requirements as fixed inputs, so the impact of a requirement decision cannot be compared against a technology assumption. Classical eVTOL UQ approaches^15,16^ propagate aerodynamic and propulsive uncertainty but again hold operational requirements constant. RIKs uniquely sit at the requirement-to-sizing interface: they are composable modules with uncertain coefficients that plug directly into the physics-based constraint loop, making requirement-origin and parameter-level uncertainty commensurable. The three contributions are: (1) RIKs embedded within a three-level uncertainty architecture that distinguishes requirement-scenario uncertainty (which RIKs are active, resolved by program management), RIK coefficient uncertainty (engineering translation models, resolved by detailed analysis), and parameter-level uncertainty (16 aleatory and epistemic variables, resolved by testing); (2) extension of the classical *T*/*W*–*W*/*S* constraint diagram into a probabilistic tool with Sobol sensitivity decomposition and an Architecture Robustness Index (ARI) for objective three-configuration comparison; and (3) a requirement sensitivity analysis that quantifies each stakeholder requirement’s design impact and translates sizing outcomes into physical vehicle geometry with uncertainty bands, producing results directly communicable to health system planners—for example, that demanding urgent delivery reduces aircraft feasibility four times more than adding cold-chain thermal management.

The framework is demonstrated through a case study of medical delivery in the Quito Metropolitan District, Ecuador. Quito extends from approximately 2,200 to 3,200 m MSL along a narrow inter-Andean valley, with surrounding terrain reaching 4,700 m at the Pichincha volcanic complex. The city’s health network spans major hospitals in the urban core and dispersed clinics across peripheral parishes at varying elevations, connected by a road network frequently disrupted by traffic congestion and landslide events. This geographic and atmospheric context makes Quito representative of the broader class of highland cities where drone-based medical delivery faces its most challenging sizing conditions. The study compares L+C, T-R, and MR architectures across five progressive requirement scenarios, from bare cargo to full urgent medical delivery. Although the case study targets medical logistics, the RIK framework and three-level uncertainty architecture apply to any eVTOL program—manned or unmanned—where non-engineering stakeholders impose requirements with design consequences: urban air mobility with noise constraints, agricultural monitoring with coverage mandates, disaster response with response-time requirements, or infrastructure inspection with sensor payload specifications.

## Results

The framework was applied to medical delivery in the Quito Metropolitan District (2,800 m MSL), comparing Lift+Cruise (L+C), Tilt-Rotor (T-R), and Multirotor (MR) architectures across five progressive requirement scenarios. All computations use $N_{MC} = 10{,}000$ Latin Hypercube samples and $N_{base} = 2{,}048$ for Sobol analysis.

### Medical requirements dominate the design space

**Fig. 1:**
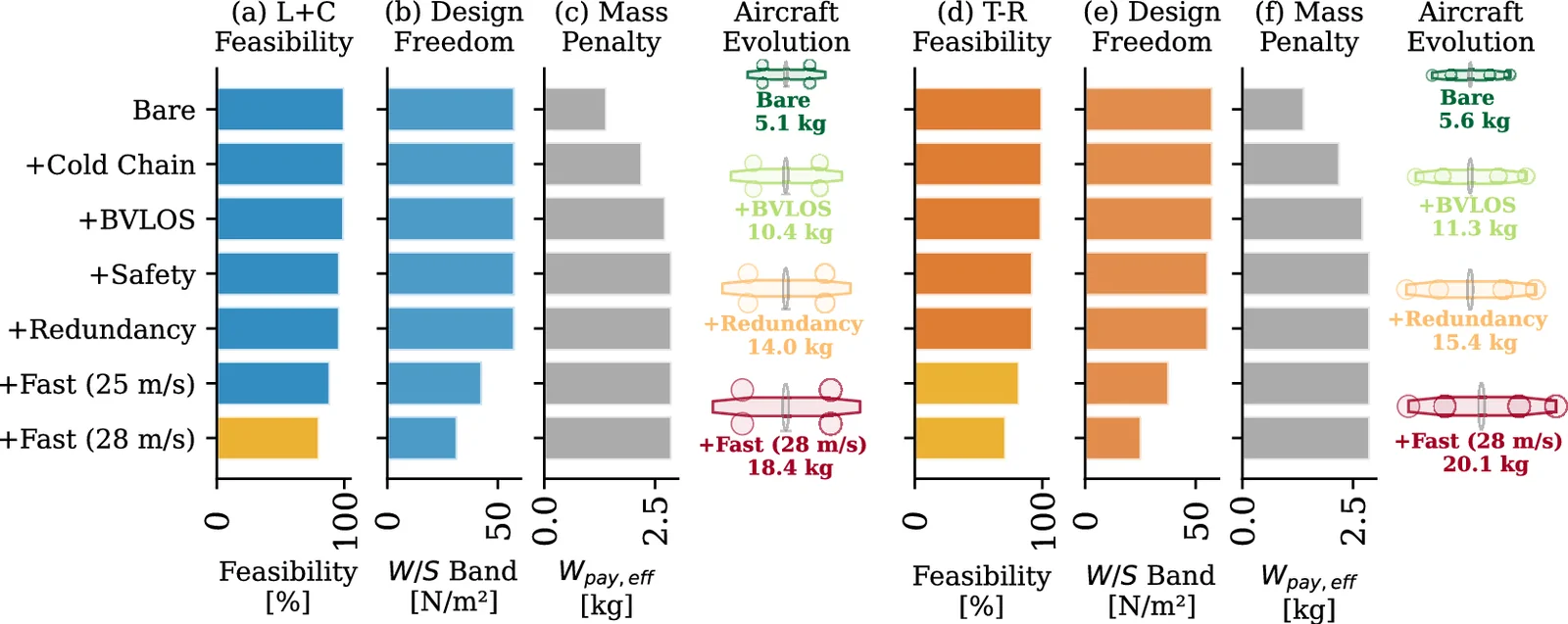
Progressive addition of medical delivery requirements and their impact on aircraft feasibility, design space width, and effective payload for both L+C and T-R architectures. Each step adds one Requirement Impact Kit.

Figure 1 presents the central result: the progressive degradation of aircraft feasibility as medical delivery requirements are added incrementally. Starting from a bare cargo UAV (97% feasibility), each step adds one Requirement Impact Kit—cold-chain compliance, beyond-visual-line-of-sight avionics, safety recovery systems, motor redundancy, and finally the speed increase demanded by urgent delivery. Table 1 provides the numerical values.

**Table 1:** Progressive RIK activation results for L+C at $h = 2{,}800$ m MSL. Each row adds one medical delivery requirement.

Requirement step	Feas. [%]	*Δ*Feas.	*W*/*S* band	$W_{pay,eff}$ [kg]
Bare	97	—	55	1.2
+Cold Chain	91	−6	50	1.8
+BVLOS	84	−7	45	2.3
+Safety	48	−36	30	2.5
+Redundancy	48	0	30	2.5
+Fast (25 m/s)	34	−14	20	2.5
+Fast (28 m/s)	24	−10	15	2.5

Three findings emerge. First, the *delivery speed* requirement is the dominant design driver: increasing cruise speed from 20 to 28 m/s costs 24 percentage points of feasibility—four times the impact of adding the entire cold-chain thermal management system (−6 percentage points). This is a health planner’s decision, not a technology parameter, yet it has far greater design consequences than any single engineering uncertainty. Second, the safety systems requirement (ballistic parachute and flight termination system) is the most impactful mass-adding requirement (−36 percentage points), driven by a feedback loop in which heavier aircraft require larger recovery hardware, which further increases weight. Third, the T-R consistently tracks 10–20 percentage points below the L+C at every requirement level. Supplementary Fig. S1 traces the corresponding migration of the design point in the *T*/*W*–*W*/*S* plane, showing how the design moves predominantly rightward toward the stall limit as requirements accumulate.

### How does the design space respond to uncertainty?

**Fig. 2:**
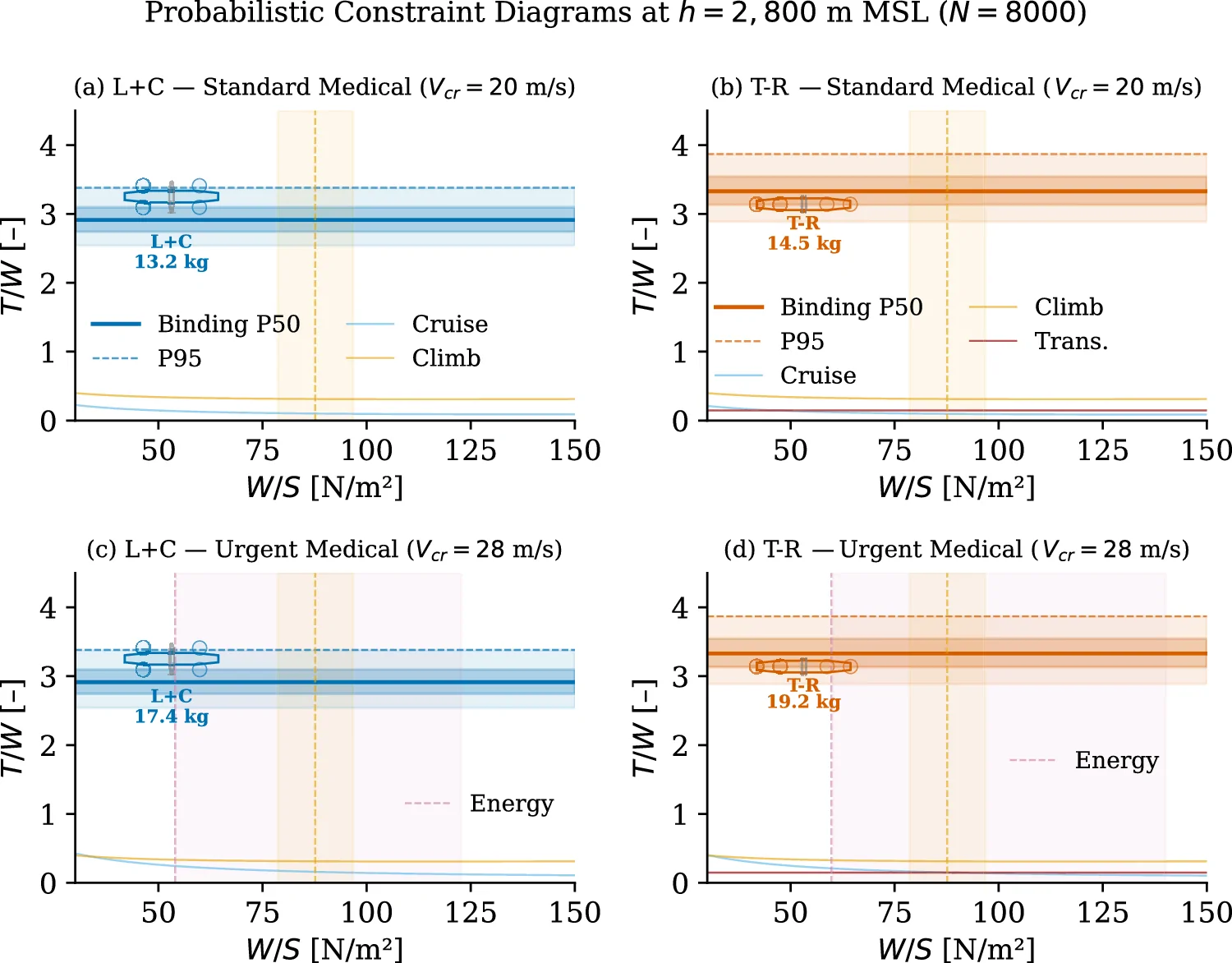
Probabilistic constraint diagrams. (**a**) L+C Standard, (**b**) T-R Standard, (**c**) L+C Urgent, (**d**) T-R Urgent. Shaded bands: percentile ranges. Vertical red band: stall limit; pink band: energy constraint.

Figure 2 presents the probabilistic constraint diagrams for four configuration–scenario combinations. Each panel shows the binding thrust-to-weight envelope as percentile bands (5th–95th and 25th–75th), with the stall limit on the right and the energy minimum on the left.

Under Standard conditions, both configurations retain approximately 53 N/m$^2$ of design freedom (the feasible *W*/*S* band between energy minimum and stall limit). Under Urgent conditions, this shrinks to 28 N/m$^2$ for the L+C and 25 N/m$^2$ for the T-R—reductions of 47% and 53% respectively (Supplementary Table S1). The T-R hover band sits 0.4 *T*/*W* units above the L+C due to the download fraction penalty, translating directly to heavier motor systems. The uncertainty spread ($\Delta T/W_{5-95} \approx 0.84$ for L+C) is wide enough to span the difference between a feasible and an infeasible design point. Supplementary Fig. S2 reveals which constraint dominates at each wing loading: under Urgent conditions, a three-way competition emerges between energy, hover, and stall constraints—the mechanism by which higher delivery speed degrades feasibility. The energy breakdown driving this competition is detailed in Supplementary Fig. S3, where the cruise energy fraction rises from 35% to 48% when $V_{cr}$ increases from 20 to 28 m/s.

### Which parameters should a development program characterize first?

**Fig. 3:**
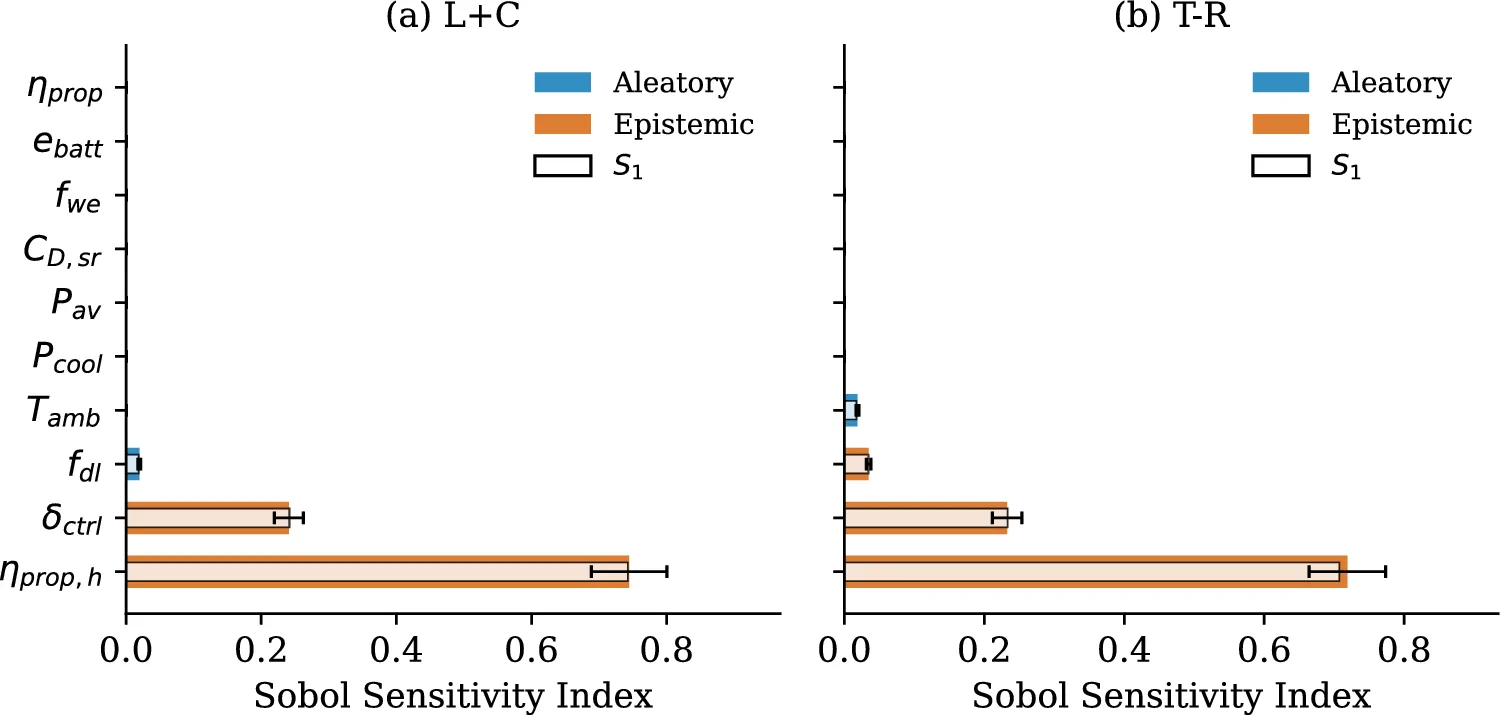
Sobol sensitivity indices for hover *T*/*W*. (**a**) L+C, (**b**) T-R. Colored bars: total-order $S_{T_i}$. White overlay: first-order $S_i$. Blue: aleatory. Orange: epistemic.

Figure 3 presents the Sobol sensitivity indices for hover *T*/*W*—the dominant constraint across most of the feasible design space. The results exhibit extreme concentration: hover propulsive efficiency ($\eta _{prop,h}$, $S_T = 0.74$) and control margin ($\delta _{ctrl}$, $S_T = 0.24$) together account for 98% of total output variance, with negligible interaction effects ($S_{T_i} - S_i < 0.01$). The T-R shows a similar structure with the download fraction $f_{dl}$ as a third-ranked parameter ($S_T = 0.035$). Critically, 98% of the total variance is epistemic (reducible): a rotor characterization campaign and site-specific turbulence measurements would recover nearly all of the design space lost to parameter-level uncertainty. By contrast, battery energy density—often cited as the primary eVTOL bottleneck—contributes less than 2% of sizing variance, because the energy constraint is dominated by cruise speed and range requirements rather than by battery performance alone. This finding is specific to the hover-dominated constraint regime at 2,800 m altitude, the 25 kg MTOW class, and the 40 km range studied here; for longer-range missions where cruise energy dominates, or for next-generation batteries with wider performance uncertainty, the battery contribution could increase.

These Sobol rankings are robust to the independence assumption: a correlation sensitivity check with rank correlation *ρ = 0.5* between $C_{D_0}$ and *e* (the most plausible correlated pair) produced no change in hover *T*/*W* variance (<0.1%), confirming that the dominant parameters ($\eta _{prop,h}$, $\delta _{ctrl}$) enter the hover constraint independently of the aerodynamic drag polar.

The value of probabilistic sizing is demonstrated by direct comparison with deterministic analysis. The deterministic design point (computed at nominal parameter values for the Standard Medical scenario) appears comfortably feasible, with stall *W/S = 87.6* N/m$^2$ and energy minimum *W/S = 30.0* N/m$^2$ providing a 57.6 N/m$^2$ band. However, when the full uncertainty is propagated, the 95%-confidence feasible band shrinks to only 7.8 N/m$^2$—a reduction of 86% that is invisible to deterministic sizing. Furthermore, conventional UQ without RIKs (propagating Level-3 parameters while fixing requirement penalties at nominal values) yields a sample feasibility of 97.5%, compared to 96.8% with the full three-level framework; while this gap appears modest for the Standard scenario, it widens considerably under Urgent conditions where the energy constraint becomes binding (Supplementary Table S6).

### Which architecture should a medical delivery program select?

**Fig. 4:**
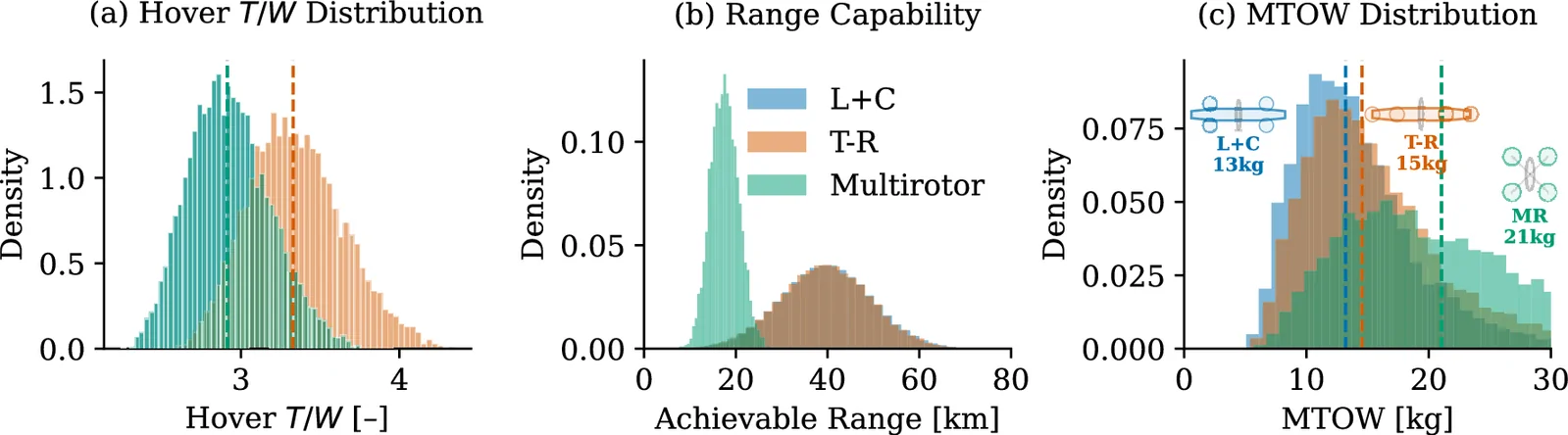
Three-configuration comparison for Standard Medical scenario at 2,800 m MSL. (**a**) Hover *T*/*W* distributions. (**b**) Achievable range (MR limited to *∼*20 km). (**c**) MTOW distributions with inset silhouettes.

Figure 4 compares all three architectures for the Standard Medical scenario. The Multirotor achieves a range of only approximately 20 km—insufficient for the 40 km round-trip that defines the standard mission—confirming that a wing is necessary for medium-range medical delivery in mountainous terrain. The L+C and T-R extend to 60–70 km range, but the L+C is consistently 10% lighter (median MTOW 13.2 kg versus 14.5 kg) and achieves higher feasibility. The Architecture Robustness Index decomposition (Supplementary Fig. S4) confirms the L+C advantage across all four metrics in both Standard and Urgent scenarios. The ARI’s multiplicative form enforces a no-compensation property appropriate for safety-critical design: any metric approaching zero drives the entire index toward zero, preventing a high score on one dimension from masking a critical weakness. The L+C ranking is robust to alternative index formulations—geometric mean, minimax, and weighted sum all produce the same architecture ordering (Supplementary Table S7).

To verify that this recommendation is not an artifact of architecture-specific parameter assumptions, we tested an optimistic T-R scenario with halved download fraction ($f_{dl} \sim \mathcal {U}(0.05, 0.10)$, representing a wing redesigned to minimize rotor-wake interaction). T-R sample feasibility increased from 93.7% to 94.1% for the Standard scenario, but the L+C (96.8%) still dominates. The architecture advantage is structural, not parametric.

### What does the aircraft look like, and where can it operate?

Table 2 summarizes the MTOW closure results at the design point ($W/S^* = 68$ N/m$^2$). For the recommended L+C under Standard Medical conditions, the median aircraft has an MTOW of 13.2 kg, a wing span of 3.9 m, and a rotor diameter of 0.59 m. The P5–P95 ranges reveal the physical consequence of requirement-origin uncertainty: the wing span varies from 3.0 to 5.4 m, meaning that the structural engineer cannot design for a single aircraft but must accommodate a family of vehicles. The full MTOW probability distributions are shown in Supplementary Fig. S5.

**Table 2:** MTOW closure results at $W/S^* = 68$ N/m$^2$, $h = 2{,}800$ m MSL. Values: P50 (P5–P95).

Config	Scenario	Feas.	MTOW [kg]	*b* [m]	*S* [m$^2$]	$d_r$ [m]
L+C	Bare	100%	5.1 (2.5–9.2)	2.4 (1.7–3.3)	0.59	0.37
T-R	Bare	100%	5.6 (2.7–10.3)	2.5 (1.8–3.4)	0.64	0.38
L+C	Standard	99%	13.2 (7.8–25.0)	3.9 (3.0–5.4)	1.52	0.59
T-R	Standard	99%	14.5 (8.4–28.7)	4.1 (3.1–5.8)	1.68	0.62
L+C	Urgent	87%	17.6 (9.1–37.5)	4.5 (3.3–6.6)	2.03	0.68
T-R	Urgent	81%	19.3 (9.9–39.6)	4.7 (3.4–6.8)	2.23	0.71

**Fig. 5:**
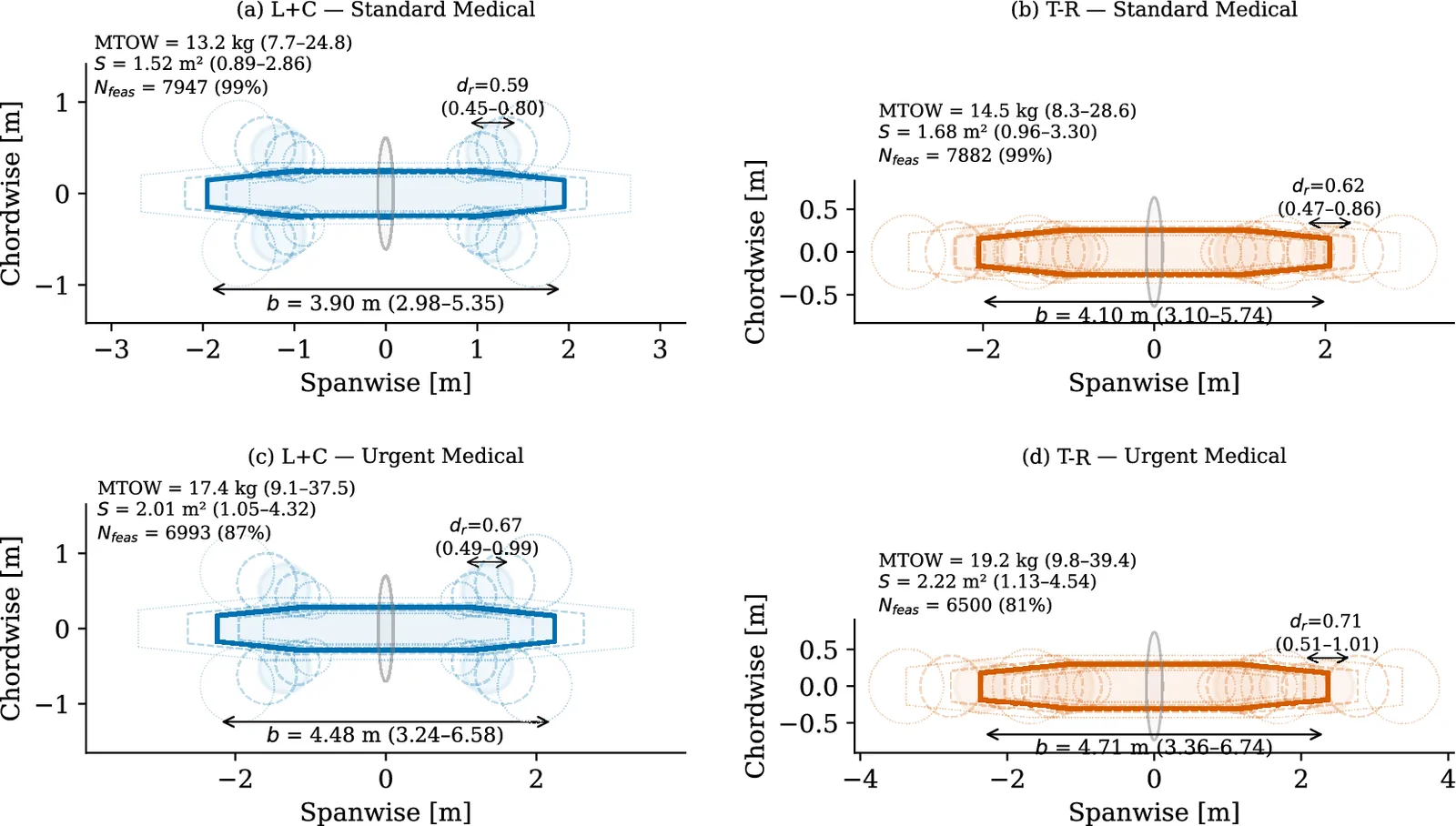
Physical vehicle geometry under uncertainty. Nested planform silhouettes at P5/P25/P50/P75/P95 for wing span, chord, and rotor diameter. (**a**) L+C Standard, (**b**) T-R Standard, (**c**) L+C Urgent, (d) T-R Urgent.

Figure 5 visualizes this geometric uncertainty through nested planform silhouettes at five percentile levels, making the sizing uncertainty tangible for non-specialist stakeholders involved in procurement and infrastructure planning.

**Fig. 6:**
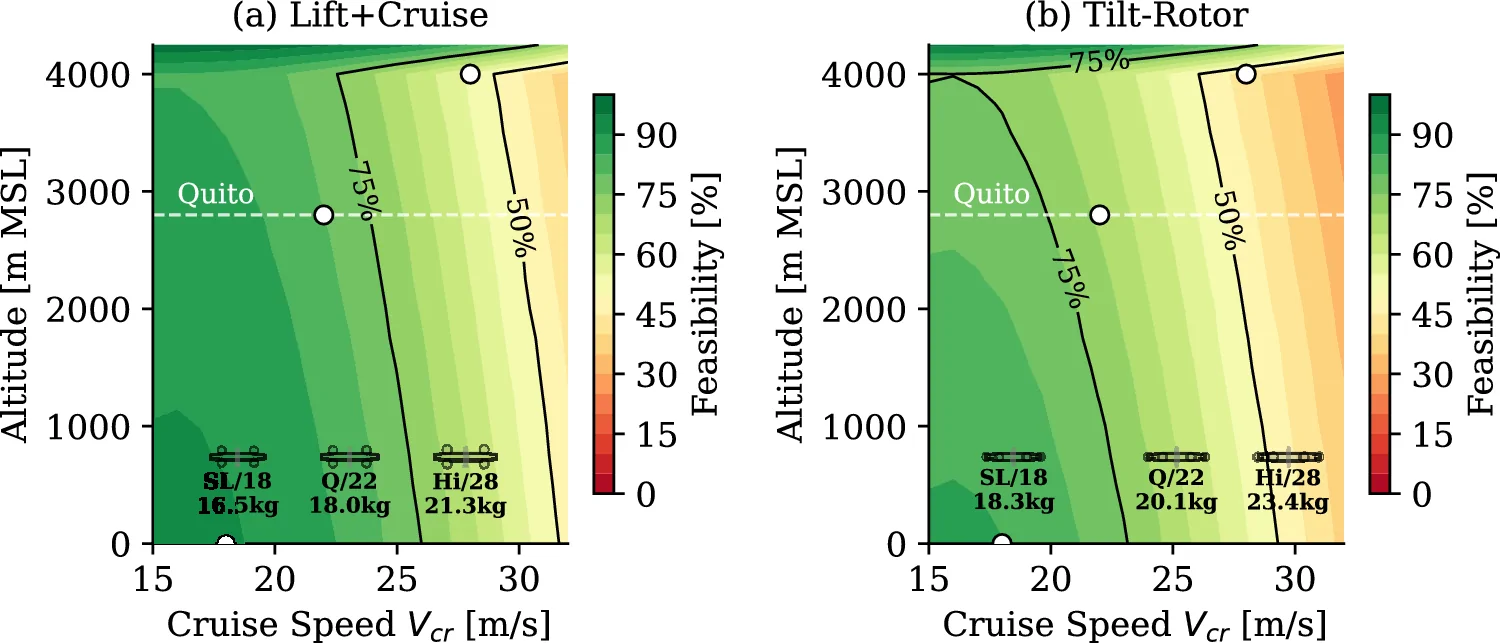
Feasibility map as a function of altitude and cruise speed for the Standard Medical scenario. (**a**) L+C, (**b**) T-R. Contour lines: 25%, 50%, 75% feasibility. Dashed line: Quito altitude.

Figure 6 provides the result most directly usable by health system planners: a feasibility map across altitude and cruise speed. At Quito’s altitude, the L+C achieves 75% feasibility up to $V_{cr} \approx 24$ m/s. A 30-minute delivery over 20 km ($V_{cr} \approx 22$ m/s) is comfortably achievable at any Andean altitude up to 4,000 m, while a 15-minute requirement ($V_{cr} \approx 33$ m/s) is infeasible above 1,000 m for either architecture. Altitude-specific feasibility curves confirming the consistent L+C advantage are provided in Supplementary Fig. S6. Multi-mission profile analysis (Supplementary Fig. S7) shows that a single L+C design achieves 95% feasibility for routine resupply but only 8% for remote highland clinics above 3,500 m, identifying an operational boundary with direct fleet planning implications.

## Discussion

The central finding of this work is that decisions made by health logistics planners—not limitations of aerospace technology—dominate the design space of medical delivery drones at high altitude. The demand for urgent delivery reduced aircraft feasibility by 24 percentage points, while adding cold-chain thermal management—a requirement imposed by physicians to preserve blood product integrity—cost only 6 points. This asymmetry has a direct practical consequence: a health ministry planning a drone delivery network in the Andes, East Africa, or the Himalayas should invest more effort in defining realistic delivery time targets than in optimizing the thermal management system, because the former dominates aircraft feasibility while the latter is comparatively inexpensive in design terms.

This finding reframes a conversation that currently occurs qualitatively between two communities that do not share a technical language. When a physician says “we need blood delivered within 30 minutes,” that specification cascades through the engineering translation chain into a cruise speed requirement that activates energy constraints, shrinks the feasible design space by half, and increases the median MTOW from 13.2 to 17.6 kg. The RIK waterfall (Fig. 1) and the feasibility heatmap (Fig. 6) make these consequences visible and quantitative. A health planner can read directly from the heatmap that a 30-minute delivery over 20 km (approximately 22 m/s cruise speed) is achievable with 75% reliability at any Andean altitude up to 4,000 m using the L+C configuration, while a 15-minute requirement (approximately 33 m/s) is infeasible above 1,000 m for either architecture.

The heatmap also demonstrates quantitative generalizability to other highland cities without re-computation. Reading directly from the altitude axis: Addis Ababa (2,355 m) achieves approximately 60% L+C feasibility at 20 m/s; La Paz (3,640 m) drops to approximately 38%; Kathmandu (1,400 m) reaches approximately 78%. These values follow the density-altitude physics encoded in the framework and confirm that the altitude–speed trade-off is portable across settings by adjusting only the reference altitude and local atmospheric statistics.

For operational decision-making, we suggest interpreting feasibility thresholds as follows: above 75%, the aircraft design is likely to close after detailed design refinement and should proceed to preliminary design; between 50% and 75%, the design is marginal and the program should plan for significant parameter characterization (rotor testing, wind measurements) before committing resources; below 50%, the program should consider relaxing requirements before proceeding, because more than half of plausible parameter combinations lead to infeasible designs. These bands provide a natural decision framework for medical logistics planners analogous to Technology Readiness Level gates in systems engineering.

Among mass-adding requirements, safety systems produced the largest single feasibility drop (−36 percentage points) due to the MTOW-coupled feedback loop. This result highlights a regulatory tension specific to medical drone programs: the requirement intended to protect people on the ground is the requirement most likely to prevent the aircraft from being feasible in the first place. At high altitude, where the available design margin is already compressed by density effects, parachute mass fractions that are manageable at sea level become prohibitive. This suggests that altitude-dependent or mission-dependent safety requirements—lighter recovery systems for operations over low-population-density terrain, for example—could substantially expand the operational envelope without compromising population safety.

The Sobol decomposition translates into a concrete development roadmap for a medical drone program. Since hover propulsive efficiency and control margin account for 98% of parameter-level variance, and both are epistemic, a rotor hover-stand campaign at representative altitude and temperature conditions combined with site-specific wind measurements at intended operating locations would recover nearly all of the design space currently lost to engineering uncertainty. By contrast, battery energy density—often cited as the primary technology bottleneck for eVTOL aircraft—contributes less than 2% of sizing variance at the conceptual stage. This does not mean that battery improvements are irrelevant to eVTOL technology broadly, but rather that at the conceptual sizing level for this mission class, the energy constraint is dominated by cruise speed and range requirements rather than by battery performance. For a medical delivery program with limited development budgets, rotor testing should be prioritized over battery qualification.

The architecture recommendation is unambiguous: the L+C achieves higher feasibility (53% versus 35% Standard, 27% versus 15% Urgent), lower MTOW (13.2 versus 14.5 kg), and superior robustness across all ARI metrics. The Multirotor, limited to approximately 20 km range, cannot serve the 40 km round-trip mission, confirming that a wing is necessary for regional medical delivery networks. This finding is relevant to health programs currently operating small multirotors for short-range delivery (e.g., vaccine distribution within 10 km): scaling to regional networks serving dispersed highland clinics will require a transition to hybrid eVTOL platforms with substantially different cost, training, and infrastructure requirements.

The multi-mission analysis reveals that a single L+C design point can serve 95% of routine resupply missions reliably but drops to 67% for urgent deliveries and only 8% for remote high-altitude clinics above 3,500 m. This heterogeneity has fleet planning implications: a highland medical drone network cannot rely on a single aircraft type for all missions. Remote clinics may require relay stations, larger airframes exceeding the 25 kg regulatory limit, or acceptance of lower delivery reliability for non-emergency supplies. The framework provides the quantitative basis for these fleet composition decisions.

The geometric uncertainty visualized in the planform bands (Fig. 5) has procurement and certification implications. A wing span range of 3.0–5.4 m (P5–P95) for the Standard Medical L+C means that final aircraft dimensions depend on which combination of uncertain parameters is realized during detailed design and testing. Certification programs should plan for this variability rather than committing to a single geometry at the conceptual stage.

Several limitations should be acknowledged, each with identifiable directional bias. First, rotor–wing interaction in hover is parameterized through the download fraction $f_{dl}$ rather than computed from blade element theory or CFD; this is conservative for the T-R (actual download may be lower with optimized wing placement) but does not capture the speed-dependent reduction during transition. Second, the T-R transition constraint uses a quasi-static force balance at a single critical tilt angle (*τ = 45*°) rather than time-resolved trajectory optimization; this may underestimate the transition *T*/*W* requirement if the actual corridor is narrower, biasing T-R feasibility slightly optimistic for this specific constraint. Third, the battery model uses constant pack-level specific energy rather than a state-of-charge-dependent voltage curve; in reality, available power decreases below approximately 20% SOC, which would tighten the energy constraint slightly—the 10% energy reserve partially compensates. Fourth, the framework does not model aeroelastic effects or wing flutter boundaries; for the span range identified (3.0–5.4 m), flutter is unlikely to bind at the speeds considered (<30 m/s) but would require verification during preliminary design. The RIK coefficient distributions reflect engineering judgment and manufacturer data rather than formal expert elicitation, and the fixed design parameters represent a single design family rather than the full trade space.

Looking forward, the framework’s limitations provide a roadmap for future research. Experimental validation via altitude flight testing, formal Delphi-method elicitation of RIK coefficients from medical and engineering panels, and higher-fidelity coupling with CFD-informed aerodynamic models (particularly for T-R transition) would refine the quantitative results. Extending the analysis to fleet-level heterogeneous missions, multi-objective optimization within the probabilistic feasible region, and validation against existing medical drone networks in East Africa will be essential for demonstrating broader applicability.

## Methods

The framework developed here translates the medical delivery requirements identified in the Introduction—cold-chain compliance, autonomous flight, safety recovery, delivery urgency—into a probabilistic aircraft sizing analysis that outputs feasibility, sensitivity rankings, and physical vehicle geometry. This section presents the complete methodology, organized around three eXtended Design Structure Matrix (XDSM) diagrams that define the computational architecture, the constraint evaluation internals, and the iterative design refinement protocol.

Supplementary Fig. S8 shows the overall computational workflow as an eXtended Design Structure Matrix (XDSM), following the notation of Lambe and Martins where blue rounded boxes denote optimization or sampling drivers, green boxes denote explicit function evaluations, and data parallelograms on the connecting arrows indicate the variables exchanged between modules. The workflow proceeds left to right, the subscript *i* denotes per-sample quantities. The Sampling Strategy module generates $N_{MC}$ Latin Hypercube samples and $N_{Sobol}$ Saltelli samples from the 16 parameter distributions (see Supplementary Fig. S11). These samples are distributed to three parallel evaluation modules: the Atmosphere Model receives ambient temperature $T_{amb,i}$ and computes the density ratio $\sigma _{\rho ,i}$; the RIK Penalty Models receive payload mass $W_{pay,i}$ and RIK coefficients and compute the effective payload $W_{pay,eff,i}$ and auxiliary power $P_{aux,i}$; and the Constraint Evaluation module receives all aerodynamic, propulsive, and energy parameters to compute the binding thrust-to-weight ratio $(T/W)_{c,i}$ at each wing loading grid point. The downstream modules perform Statistical Processing (extracting percentile boundaries), Sobol Sensitivity Analysis (computing first-order $S_i$ and total-order $S_{T_i}$ indices), and Robustness Assessment (computing the feasible area $\mathcal {A}_{0.95}$, contraction ratio *Γ*, and Architecture Robustness Index ARI), culminating in the design point $(T/W^*,\, W/S^*)$. The full XDSM diagrams for the framework, the iterative design refinement protocol, and the constraint evaluation module are provided in Supplementary Figs. S8–S10.

The iterative design refinement protocol (Supplementary Fig. S9) connects the probabilistic constraint analysis to preliminary design and testing. The feedback path from the Testing and Characterization block back to Uncertainty Specification represents the key iterative loop: as epistemic parameters are characterized through rotor testing, wind tunnel campaigns, or battery cycling experiments, the distributions are updated with refined means and reduced standard deviations, and the entire analysis is re-executed to quantify the design space recovered by uncertainty reduction.

### Requirement impact kits and three-level uncertainty architecture

The central methodological innovation is the formalization of how qualitative medical delivery requirements—originating from physicians, aviation regulators, and health logistics planners—enter the aircraft sizing loop as uncertain quantitative penalties. A Requirement Impact Kit (RIK) is defined as a modular unit that translates a single qualitative requirement into quantitative design penalties:

1 $$\begin{aligned} \text {RIK}_j = \left\{ \Delta W_j(W_{pay}, W_{MTO}),\; P_j,\; \mathcal {C}_j\right\} \end{aligned}$$

where $\Delta W_j$ is a mass penalty model that may depend on payload mass or MTOW, $P_j$ is a continuous electrical power draw during flight, and $\mathcal {C}_j$ is a set of constraint modifications such as minimum rotor count or cruise speed override. The mass penalty takes the general linear form:

2 $$\begin{aligned} \Delta W_j = \tilde{a}_j + \tilde{b}_j \cdot W_{pay} + \tilde{c}_j \cdot W_{MTO} \end{aligned}$$

where tildes denote random variables whose distributions reflect the uncertainty in the engineering translation process. Each coefficient has a distinct physical meaning accessible to non-aerospace readers: $\tilde{a}_j$ is a fixed mass penalty independent of aircraft or payload size (e.g., the mass of an avionics box or a parachute deployment mechanism); $\tilde{b}_j$ is a payload-proportional penalty (e.g., insulation material that scales with the volume of blood products being transported); and $\tilde{c}_j$ is an MTOW-proportional penalty (e.g., a parachute canopy whose area must scale with the aircraft weight it decelerates, creating a feedback loop). The power term $P_j$ represents continuous electrical power drawn from the battery during flight (e.g., a Peltier cooler maintaining blood temperature at 1–6°C). Not every RIK uses all three mass terms; most have at most two non-zero coefficients. Supplementary Table S2 defines the seven RIKs developed for the medical delivery case study, with tildes ($\tilde{\cdot }$) denoting uncertain engineering coefficients. Each RIK traces to a specific stakeholder requirement. The Baseline RIK represents the minimum avionics mass for visual-line-of-sight operations. The cold-chain RIKs (CC-Passive and CC-Active) translate the physician’s requirement that blood products remain at 1–6°C: passive systems use phase-change materials with mass scaling linearly with payload, while active systems add a Peltier cooler with both a fixed container mass and a payload-proportional insulation term. The BVLOS Avionics RIK translates the regulator’s requirement for autonomous beyond-visual-line-of-sight operations into the mass and power of the communication and navigation hardware. The Safety RIK translates the aviation authority’s mandate for safe flight termination into a ballistic parachute recovery system whose mass scales with MTOW through the fraction $\tilde{c}$, creating the feedback loop discussed in the Introduction. The Redundancy RIK translates the motor-failure-tolerance requirement into an increased motor count, while the Fast Delivery RIK translates the health planner’s urgency requirement into a cruise speed override of 28 m/s.

Supplementary Table S3 groups these RIKs into five requirement scenarios representing progressively more demanding medical delivery program definitions. Scenario S0 represents a bare cargo UAV with no medical-specific requirements—the baseline against which all medical requirements are measured. S1 adds passive cold-chain compliance, typical of short-range deliveries where transit times are brief enough that phase-change materials suffice. S2 represents a full standard medical mission with active cooling, autonomous flight, safety systems, and quad-motor redundancy—the minimum configuration a health ministry would likely approve for routine blood delivery operations. S3 adds the urgency requirement, increasing cruise speed from 20 to 28 m/s to support emergency deliveries where every minute of delay carries clinical risk. S4 represents the most demanding combination, adding hexacopter redundancy to the urgent scenario. The progressive structure enables the requirement sensitivity analysis presented in the Results section, where the feasibility impact of each medical requirement addition is isolated.

The framework propagates uncertainty at three hierarchical levels. *Level 1* addresses requirement-scenario uncertainty: which RIKs are active. This is a discrete choice resolved by stakeholder and management decisions during the program definition phase—for instance, the decision to require active cold-chain compliance versus passive insulation, or to mandate 28 m/s cruise speed for urgent delivery versus 20 m/s for routine operations. The framework evaluates all five scenarios (Supplementary Table S3), quantifying the feasibility consequence of each requirement decision. *Level 2* addresses the uncertain engineering coefficients within each active RIK. Given that the cold-chain RIK is active, for example, the insulation scaling coefficient $\tilde{b}$ still carries uncertainty reflecting the range of defensible thermal system designs. These are epistemic and would be narrowed by a detailed thermal analysis. *Level 3* addresses the 16 classical sizing parameters (aerodynamic, propulsive, energy, environmental), which combine aleatory variability (weather, payload mass) with epistemic uncertainty (component performance). These are propagated via Latin Hypercube Monte Carlo simulation with Sobol sensitivity decomposition. The three levels are not interchangeable: Level 1 is resolved by program management (health ministry and aviation authority decisions), Level 2 by engineering analysis (detailed thermal, structural, and systems design), and Level 3 by testing and operations (rotor characterization, wind measurements, flight trials). Statistical independence is assumed among all parameters within each level, following the standard recommendation for Sobol analysis^19^. Weak correlations may exist in practice—for example, $C_{D_0}$ and Oswald efficiency *e* are physically linked through wetted area, and ambient temperature and wind speed may co-vary through weather systems. At the conceptual stage, the independence assumption is conservative: it produces wider uncertainty bands than correlated sampling, so our feasibility estimates represent lower bounds. We verify this through a correlation sensitivity check reported in the Results section.

The practical consequence of this layered structure is illustrated in Supplementary Fig. S12, which compares the distributions of three key derived quantities under single-level versus two-level uncertainty propagation for the Standard Medical scenario (S2). The effective payload distribution broadens substantially when RIK coefficients are uncertain (Levels 2+3 combined) compared to fixed nominal values (Level 3 only), because the cold-chain insulation coefficient and the parachute MTOW fraction contribute variance that is invisible to classical single-level UQ. The effective empty weight fraction shifts rightward by approximately 0.06, meaning that 6% of MTOW is consumed by requirement-imposed systems.

### Case study definition and uncertain parameters

The case study considers medical delivery UAVs serving the health facility network of the Quito Metropolitan District, Ecuador. Quito’s public health system includes major hospitals in the urban core and over 50 primary care clinics dispersed across parishes at elevations ranging from 2,200 to 3,200 m MSL. Ground-based delivery between these facilities requires navigating a congested road network through a narrow inter-Andean valley, with typical transit times of 45–90 minutes for one-way distances of 15–40 km. The reference altitude of $h_{ref} = 2{,}800$ m MSL represents the operational center of gravity of this network. The atmospheric model follows the International Standard Atmosphere (ISA) with temperature deviation^12^. The density ratio is computed by separating the pressure and temperature contributions:

3 $$\begin{aligned} \sigma _\rho = \frac{\rho }{\rho _{SL}} = \left( \frac{T_{ISA}(h)}{T_{SL}}\right) ^{\!\frac{g}{RL}-1} \cdot \frac{T_{ISA}(h)}{T_{ISA}(h) + \Delta T} \end{aligned}$$

where $T_{ISA}(h) = T_{SL} - Lh$ is the ISA temperature profile with lapse rate *L = 0.0065* K/m, sea-level temperature $T_{SL} = 288.15$ K, gravitational acceleration *g = 9.807* m/s$^2$, and specific gas constant *R = 287.06* J/(kg*·*K). The deviation $\Delta T = T_{amb} - T_{ISA}(h)$ is computed from the sampled ambient temperature, coupling the stochastic temperature field to the density ratio. At the reference altitude with $T_{amb} = 15$°C, the model yields $\sigma _\rho \approx 0.71$, which is consistent with meteorological observations for Quito.

#### Uncertain parameter selection and distribution justification

Sixteen uncertain parameters are propagated through the analysis. Supplementary Table S4 lists each parameter with its distribution, bounds, classification, and the rationale for the distribution choice. The classification follows standard UQ practice: aleatory parameters represent irreducible variability inherent in the operating environment, while epistemic parameters represent reducible uncertainty from incomplete knowledge of system characteristics^26^.

The four aleatory parameters capture operational variability that cannot be reduced by design improvements. Payload mass is modeled as uniform between 0.5 kg (a single blood bag of approximately 0.45 kg plus minimal packaging) and 2.0 kg (multi-unit blood delivery with insulated container), reflecting the full range of medical payloads documented in drone delivery trials^6,8^. Mission range follows a normal distribution centered at 40 km with a standard deviation of 10 km, derived from the geographic distribution of health facilities around Quito where one-way distances span approximately 10–60 km. Ambient temperature is modeled as $\mathcal {N}(15, 7)$°C based on Quito’s annual climate, which ranges from near-freezing at night during cold months to approximately 30 °C during daytime in warm months; the normal distribution captures the concentration of operations during daytime hours while allowing for extreme conditions in the tails. Wind speed follows a Weibull distribution with scale parameter *λ = 5* m/s and shape parameter *k = 2* (a Rayleigh distribution), which is the standard model for surface wind speeds^19^ and is consistent with meteorological records for the inter-Andean valley, where median wind speeds of 4–6 m/s are typical with occasional gusts exceeding 15 m/s.

The twelve epistemic parameters reflect incomplete knowledge of component performance at the conceptual design stage. Triangular distributions are used when a most likely value and physical bounds are known but the detailed shape of the distribution is not—this is the appropriate choice for expert-elicited engineering estimates^19^. Uniform distributions are used when only the credible range is known without a clear mode, representing maximum ignorance within bounded support. Normal distributions are used for parameters where a nominal value and dispersion are established from historical data or manufacturer specifications. Supplementary Fig. S11 displays all 16 distributions with their theoretical probability density functions overlaid on 20,000 LHS samples; among the epistemic parameters, the hover propulsive efficiency $\eta _{prop,h}$ and the control margin $\delta _{ctrl}$ have the broadest relative ranges, foreshadowing their dominant role in the Sobol sensitivity analysis.

The battery pack energy density distribution merits particular attention. Cell-level energy densities for current lithium-polymer cells range from 250 to 300 Wh/kg, but pack-level performance is significantly lower due to cell-to-pack knockdown factors of 30–40% that account for packaging, bus bars, battery management systems, and thermal management hardware^27^. Furthermore, mid-life capacity fade of 10–15% at 300 cycles is typical for small UAV battery packs. The distribution $\mathcal {N}(180, 20)$ Wh/kg therefore represents the realistic operational energy density after accounting for both integration losses and aging, consistent with the multifidelity battery UQ methodology proposed by the Vertical Flight Society (VFS)^17^.

The hover propulsive efficiency $\eta _{prop,h}$ is modeled as Tri(0.45, 0.55, 0.65) based on rotor figure-of-merit data. Small-scale rotors (diameter *< 1* m) operating at disc loadings of 100–150 N/m$^2$ typically achieve figures of merit between 0.45 and 0.65, with the lower values corresponding to low-Reynolds-number blade sections and the upper values to optimized blade designs tested in controlled conditions^28^. The control margin $\delta _{ctrl}$, modeled as $\mathcal {U}(0.25, 0.45)$, represents the fractional excess thrust required above hover to maintain attitude control in turbulence; the lower bound corresponds to calm conditions where only attitude stabilization is needed, while the upper bound reflects operations in gusty Andean terrain requiring substantial control authority for gust rejection.

#### Configuration definitions

Three eVTOL architectures are compared. The Lift+Cruise (L+C) uses four dedicated lift rotors for hover and a separate pusher propeller for cruise. During forward flight the lift rotors are stopped, incurring a drag penalty quantified by the stopped-rotor drag coefficient $C_{D,sr} \sim \mathcal {U}(0.3, 0.8)$—the wide range reflects the strong sensitivity to blade alignment angle documented by Wang et al.^29^, who measured drag increases of 30–40% depending on whether blades are feathered parallel or perpendicular to the flow. The Tilt-Rotor (T-R) tilts the same rotors between vertical and horizontal orientations, achieving cleaner cruise aerodynamics but introducing a download penalty in hover of 10–15% of total thrust^28^ and a transition corridor where both rotor tilt and aerodynamic lift must be carefully managed^30^. The pure Multirotor (MR) uses rotors exclusively in all flight phases, serving as a range-limited baseline to quantify the penalty of not having a wing.

Fixed design parameters common to all winged configurations are summarized in Supplementary Table S5. The stall speed of 12 m/s ensures safe low-speed flight for a small UAV and is consistent with certified light UAV designs in the 10–25 kg class. The wing aspect ratio of 8 balances cruise efficiency against structural weight for the expected wing span range of 2–6 m. The disc loading of 120 N/m$^2$ is representative of small eVTOL rotors, where lower values would require impractically large rotor diameters and higher values would degrade hover efficiency. The MTOW limit of 25 kg corresponds to the regulatory boundary for many small UAV operating categories, including Ecuador’s DGAC regulations. The hover time of 180 s accounts for takeoff from a hospital dispatch point, landing at a clinic with possible rooftop or courtyard approach, and a brief loiter margin for package handoff. The altitude gain of 300 m represents the typical terrain clearance required when flying between Quito’s valley floor and elevated peripheral parishes. Cruise speed is scenario-dependent: 20 m/s for standard scenarios and 28 m/s for urgent delivery, where the speed increase reflects the health planner’s requirement to halve delivery time from approximately 30 to 15 minutes over a 20 km one-way distance.

### Constraint analysis formulation

The constraint analysis maps performance requirements onto the *T*/*W* versus *W*/*S* design plane, where each constraint produces either a lower bound on *T*/*W* at a given *W*/*S* (thrust constraints) or an upper or lower bound on *W*/*S* independent of *T*/*W* (stall and energy constraints). The internal data flow of the constraint evaluation module is detailed in Supplementary Fig. S10. The Configuration Selector at the top activates architecture-specific terms: the stopped-rotor drag coefficient $\Delta C_{D,sr}$ enters the cruise constraint only for the L+C, the download fraction $f_{dl}$ enters the hover constraint only for the T-R, and the tilt angle *τ* and transition lift coefficient $C_{L_{trans}}$ activate the transition constraint only for the T-R. The Aerodynamic Model computes the drag polar coefficients $C_D(C_L)$ and induced drag factor $K = 1/(\pi \,e\,AR)$, which feed the cruise, climb, and energy constraints. All six constraint outputs converge at the Feasible Region Intersection solver, which computes the combined feasible region $\mathcal {F}_C$ for configuration *C* by taking the intersection of all individual constraint-satisfying regions.

#### Stall constraint

The stall constraint establishes the maximum wing loading by requiring that the aircraft can sustain flight at the minimum speed $V_{stall}$ without exceeding the maximum lift coefficient. From the definition of lift at stall^12^:

4 $$\begin{aligned} (W/S)_{stall} = \tfrac{1}{2}\,\rho _{SL}\,\sigma _\rho \,V_{stall}^2\,C_{L_{max}} \end{aligned}$$

This appears as a vertical boundary in the *T*/*W*–*W*/*S* plane. At the reference altitude with $\sigma _\rho \approx 0.71$ and a nominal $C_{L_{max}} = 1.4$, the stall limit is approximately 86 N/m$^2$, which is 28% below the sea-level value of 119 N/m$^2$. Uncertainty in $C_{L_{max}}$ (from airfoil selection) and $\sigma _\rho$ (from ambient temperature) propagates directly to this boundary, producing a band of approximately 20 N/m$^2$ width between the 5th and 95th percentiles.

#### Cruise and climb constraints

The cruise constraint requires sufficient thrust for steady level flight at $V_{cr}$. Starting from the equilibrium of thrust and drag with the parabolic drag polar $C_D = C_{D_0} + K\,C_L^2$ where the induced drag factor is $K = 1/(\pi \,e\,AR)$^11^, and substituting $C_L = (W/S)/q_{cr}$ where $q_{cr} = \tfrac{1}{2}\rho \,V_{cr}^2$ is the cruise dynamic pressure, the required thrust-to-weight ratio is:

5 $$\begin{aligned} \left( \frac{T}{W}\right) _{\!cruise} = \frac{q_{cr}\,(C_{D_0} + \Delta C_{D,sr})}{W/S} + \frac{K\,(W/S)}{q_{cr}} \end{aligned}$$

The first term represents zero-lift drag and decreases with increasing *W*/*S* (lighter wing loading means more wetted area per unit weight), while the second term represents induced drag and increases with *W*/*S*. This produces the classical bucket-shaped curve in the design space. For the L+C configuration, the stopped-rotor drag increment $\Delta C_{D,sr}$ adds approximately 0.004 multiplied by the sampled $C_{D,sr}$ coefficient, accounting for the four lift rotors exposed to the freestream. For the T-R and MR, this increment is zero.

The climb constraint adds the potential energy rate term. From the energy-based derivation^12^, the required thrust-to-weight ratio at the best climb speed becomes $(T/W)_{climb} = RoC/V_{climb} + (T/W)_{cruise,V_{climb}}$, where the first term accounts for the rate of altitude gain and the second for drag at the climb speed. The service ceiling constraint evaluates the same expression at the ceiling density ratio $\sigma _{ceil}$ with *RoC = 0.5* m/s (the standard definition of service ceiling).

#### Hover constraint

The hover constraint is independent of wing loading and appears as a horizontal line in the design space. The required thrust must overcome aircraft weight, provide a control margin for attitude stabilization and gust rejection, and account for the density-dependent scaling of rotor efficiency. For the L+C with dedicated lift rotors, the required thrust-to-weight ratio is derived from momentum theory^28^:

6 $$\begin{aligned} \left( \frac{T}{W}\right) _{\!hover,\,L+C} \ge \frac{1 + \delta _{ctrl}}{\eta _{prop,h}\,\sqrt{\sigma _\rho }} \end{aligned}$$

The numerator $(1 + \delta _{ctrl})$ accounts for the control margin required beyond trimmed hover. The denominator $\eta _{prop,h}\,\sqrt{\sigma _\rho }$ combines the hover propulsive efficiency (rotor figure of merit) with the density-altitude scaling: lower density requires higher induced velocity for the same thrust, scaling hover power as $P_h \propto T^{3/2}/\sqrt{\rho }$, which translates to a $1/\sqrt{\sigma _\rho }$ factor in the *T*/*W* requirement.

For the Tilt-Rotor, the wing immersed in rotor downwash creates a download force that opposes lift. This download fraction $f_{dl}$, typically 10–15% of rotor thrust^28^, requires additional installed thrust:

7 $$\begin{aligned} \left( \frac{T}{W}\right) _{\!hover,\,TR} \ge \frac{1 + \delta _{ctrl}}{(1 - f_{dl})\,\eta _{prop,h}\,\sqrt{\sigma _\rho }} \end{aligned}$$

The MR hover constraint is identical to Eq. (6) since there is no wing to create download.

#### Transition constraint (Tilt-Rotor only)

At intermediate tilt angle *τ* during the transition from hover to cruise, the T-R must simultaneously support its weight vertically and overcome drag horizontally. The critical condition occurs near *τ = 45*°, where the rotor thrust vector is equally divided between lift and forward force. At the corresponding transition speed $V_{trans} = \sqrt{2(W/S)/(\rho \,C_{L_{trans}})}$ with $C_{L_{trans}} = 0.7\,C_{L_{max}}$ providing margin below stall^30^, the combined force balance yields a diagonal constraint in the *T*/*W*–*W*/*S* plane. This constraint does not appear for L+C or MR architectures, and its presence represents an additional design restriction unique to tilt-rotor configurations.

#### Energy constraint

Beyond instantaneous force-balance constraints, the aircraft must carry sufficient battery energy to complete the entire mission profile. This energy constraint is novel in the present framework because it creates a *minimum* feasible wing loading—a lower bound on *W*/*S*—that competes with the stall-imposed upper bound. The physical mechanism is as follows: at low wing loading the cruise lift coefficient is low, placing the operating point on the high-drag side of the drag polar where parasite drag dominates. This poor cruise *L*/*D* demands more energy for the same range, requiring a larger battery that may exceed the available mass budget. The energy constraint therefore penalizes low *W*/*S*, creating a feasible band between the energy minimum and the stall maximum whose width quantifies the available design freedom. This mechanism is central to the medical delivery problem: when a health planner increases the cruise speed requirement to reduce delivery time, the energy minimum *W*/*S* rises, the feasible band narrows, and more Monte Carlo samples become infeasible—the direct link between a clinical urgency demand and aircraft sizing failure.

The mission energy budget is decomposed into four segments. The hover energy per unit aircraft weight is computed from momentum theory^28^ as $e_{hover} = (T/W)_{ops} \cdot v_i / \eta _{prop,h} \cdot t_{hover}$, where $(T/W)_{ops} \approx 1.1$ is the operational hover thrust ratio. This operational value is deliberately lower than the installed design *T*/*W* from Eqs. (6)–(7), because calm-air hover does not require the full control margin $\delta _{ctrl}$ that is sized for gust rejection. The rotor induced velocity $v_i = \sqrt{(T/W)_{ops} \cdot DL / (2\rho )}$ follows from actuator disc theory. The cruise energy per unit weight is $e_{cruise} = R / (\eta _{prop} \cdot L/D)$, where *L*/*D* depends on wing loading through the parabolic drag polar. The climb energy per unit weight is $e_{climb} = g \cdot \Delta h / (\eta _{prop} \cdot \eta _{climb})$, where the climb efficiency factor $\eta _{climb} = 0.85$ accounts for the additional parasite and induced drag during the climb segment beyond the pure potential energy cost. The auxiliary energy covers all RIK-imposed electrical loads integrated over the mission duration: $E_{aux} = P_{aux} \cdot t_{mission}$, where $t_{mission} = R/V_{cr} + t_{hover} + \Delta h / RoC$ is the total mission time.

The available battery mass fraction is the residual after subtracting structural and payload fractions from unity:

8 $$\begin{aligned} f_{batt} = 1 - f_{we,eff} - \frac{W_{pay,eff}}{W_{MTO,max}} \end{aligned}$$

where the effective empty weight fraction $f_{we,eff}$ is defined in Eq. (11). The total available battery energy is then $E_{avail} = f_{batt} \cdot W_{MTO,max} \cdot e_{batt}$, expressed in joules using $e_{batt}$ in J/kg. After allocating energy for hover, climb, auxiliary systems, and the 10% reserve, the remaining cruise energy budget determines the minimum acceptable cruise *L*/*D*:

9 $$\begin{aligned} (L/D)_{req} = \frac{W_{MTO,max}\,g \cdot R \cdot (1+f_{res})}{\eta _{prop} \cdot E_{cruise,budget}} \end{aligned}$$

where $E_{cruise,budget} = E_{avail} - (e_{hover} + e_{climb})(1+f_{res}) \cdot W_{MTO,max} \cdot g - P_{aux} \cdot t_{mission}$ is the battery energy remaining after all non-cruise allocations. To find the minimum *W*/*S* that achieves this *L*/*D*, the relationship $L/D = C_L / (C_{D_0} + K\,C_L^2)$ is rearranged with $C_L = (W/S)/q_{cr}$, yielding the quadratic $K \cdot (L/D)_{req} \cdot C_L^2 - C_L + C_{D_0} \cdot (L/D)_{req} = 0$. When the discriminant of this quadratic is positive, the lower root gives the minimum lift coefficient for which the cruise *L*/*D* is sufficient, and the minimum wing loading is $(W/S)_{min} = C_{L,low} \cdot q_{cr}$. When the discriminant is negative, the required *L*/*D* exceeds the maximum achievable *L*/*D* of the drag polar, and the mission is infeasible regardless of *W*/*S*.

Configuration-specific mass penalties enter through $f_{we,eff}$. The T-R includes an additional 2.5% of MTOW for the tilt mechanism (actuators, bearings, structural reinforcement), and the L+C includes 1% for rotor stow alignment hardware. An incremental motor mass fraction $f_{motor} = 0.015 \cdot \max (T/W_{design} - 1,\, 0)$ couples the hover *T*/*W* requirement back into the energy constraint: architectures requiring higher installed *T*/*W* (such as the T-R with its download penalty) incur a heavier motor system, reducing the available battery fraction and tightening the energy constraint. This coupling ensures that the hover and energy constraints do not operate independently but interact through the weight budget.

### Design driver penalty integration via RIKs

For a given requirement scenario $\mathcal {S}$ (e.g., Standard Medical, which activates cold-chain, BVLOS, safety, and redundancy RIKs simultaneously), the effective payload mass and adjusted empty weight fraction that enter the energy constraint are:

10 $$\begin{aligned} W_{pay,eff} = W_{cargo} + \sum _{j \in \mathcal {J}_\mathcal {S}} \bigl (\tilde{a}_j + \tilde{b}_j\,W_{cargo}\bigr ) \end{aligned}$$

11 $$\begin{aligned} f_{we,eff} = f_{we} + \sum _{j \in \mathcal {J}_\mathcal {S}} \tilde{c}_j + f_{motor}(T/W) + \Delta f_{config} \end{aligned}$$

where the RIK coefficients are sampled independently for each Monte Carlo realization. The active cold-chain system contributes a fixed container mass ($\tilde{a}_{th} \sim \mathcal {N}(0.4, \text {CV}=0.15)$ kg, representing the Peltier module, heat sink, and container shell) plus a payload-proportional term ($\tilde{b}_{th} \sim \mathcal {U}(0.2, 0.45)$ kg/kg, capturing the insulation and phase-change material that scales with cargo volume)^31^. Its power draw $P_{cool} \sim \text {Tri}(15, 22, 30)$ W spans the range from well-insulated containers in mild conditions to poorly insulated containers in warm ambient. The ballistic parachute system mass scales with MTOW through $\tilde{c}_s \sim \mathcal {U}(0.04, 0.08)$, where the lower bound represents lightweight spring-deployed systems and the upper bound represents pyrotechnic systems with redundant deployment. This MTOW-coupled term creates a positive feedback loop: heavier aircraft require larger parachutes, which increase MTOW, requiring still larger parachutes. The BVLOS avionics mass $\tilde{a}_{av} \sim \mathcal {U}(0.35, 0.70)$ kg reflects the range between a minimal suite (single RTK-GNSS receiver, basic IMU, one LTE modem: approximately 0.35 kg) and a high-integrity system (dual GNSS, tactical-grade IMU, redundant communications, ADS-B transponder: approximately 0.70 kg).

### Uncertainty propagation and sensitivity analysis

Uncertainty propagation uses Latin Hypercube Sampling (LHS) with $N_{MC} = 10{,}000$ samples. A convergence study (Supplementary Fig. S13) confirms that the coefficient of variation of the feasibility estimate drops below 2% at $N = 5{,}000$ and below 1% at $N = 10{,}000$, while the 95th-percentile *T*/*W* boundary is stable to within 0.5% for $N> 5{,}000$. The LHS stratification provides variance reduction over simple random sampling, ensuring stable tail percentile estimates at moderate sample sizes^19^. For each sample, the procedure draws values for all 16 Level-3 parameters plus the active Level-2 RIK coefficients, computes the density ratio via Eq. (3), assembles the effective payload and weight penalties via Eqs. (10)–(11), evaluates all constraint equations across a *W*/*S* grid from 30 to 150 N/m$^2$ in steps of 2 N/m$^2$, and records the binding (maximum) *T*/*W* envelope at each grid point. Algorithm 1 formalizes this workflow.

Variance-based global sensitivity analysis decomposes the output variance into contributions from individual parameters and their interactions using Sobol indices^18^. The first-order index $S_i$ measures the fraction of output variance attributable to parameter $X_i$ independently, while the total-order index $S_{T_i}$ includes all interactions involving $X_i$. Their difference $S_{T_i} - S_i$ quantifies interaction strength. A parameter with high $S_{T_i}$ but low $S_i$ has influence primarily through interactions with other parameters, while $S_{T_i} \approx S_i$ indicates additive behavior. The indices are estimated using the Saltelli sampling scheme with $N_{base} = 2{,}048$, which generates $N_{base}(2d+2)$ total model evaluations for *d = 16* parameters^19^, yielding approximately 69,000 constraint evaluations. Bootstrap confidence intervals are computed from 1,000 replicates. The implementation uses the SALib Python library^32^.

**Algorithm 1:**
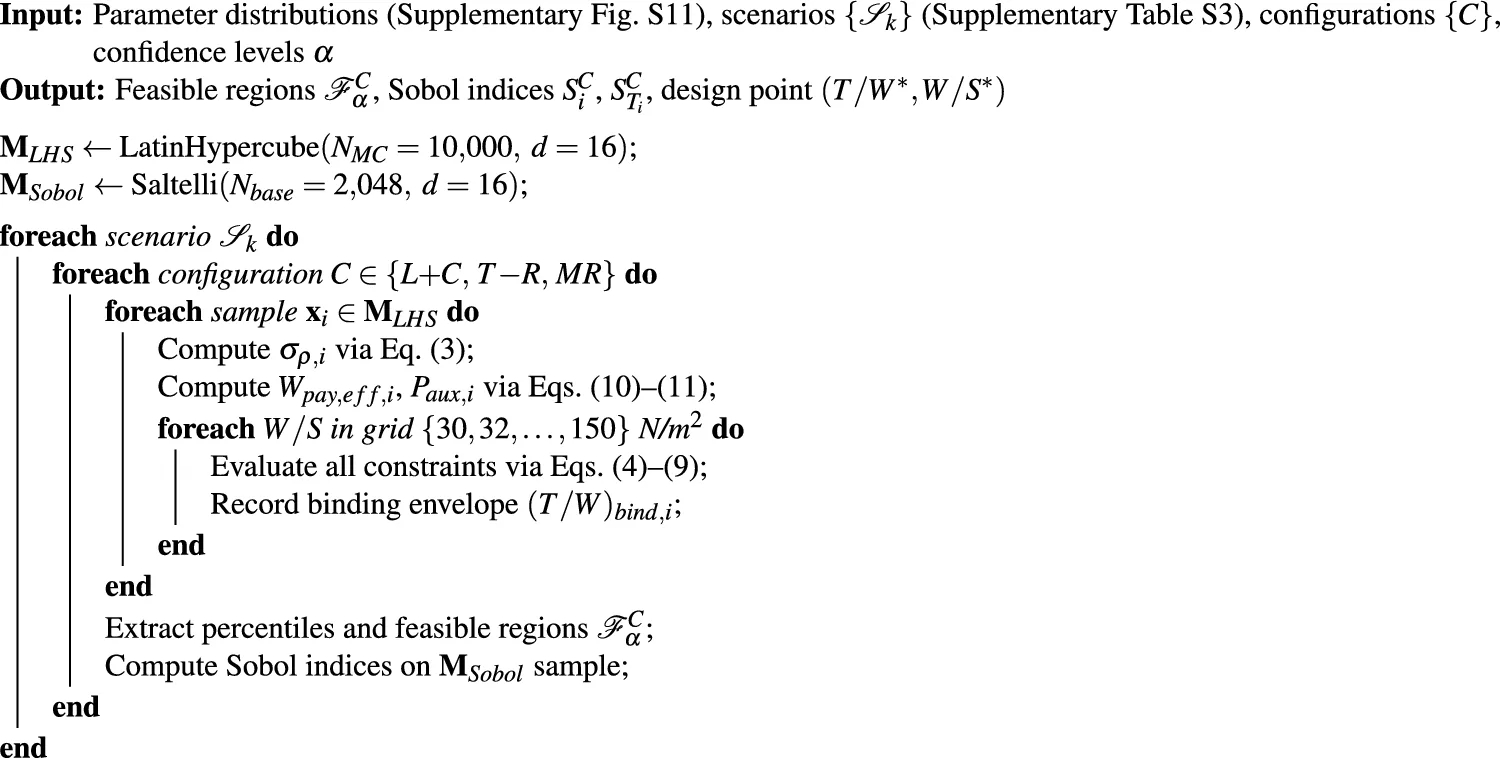
Probabilistic constraint analysis and sensitivity decomposition.

### Robustness assessment and design point selection

Architecture robustness is quantified through the Architecture Robustness Index (ARI), which provides a single scalar metric for comparing L+C, T-R, and MR configurations under the full uncertainty specification—the quantitative basis for the architecture recommendation that a medical delivery program needs before committing to a development path. The ARI combines three complementary metrics. The first is the feasible design space area $\mathcal {A}_{0.95}$ at the 95% confidence level, computed by integrating the 95th-percentile binding *T*/*W* boundary over the feasible *W*/*S* range. Larger $\mathcal {A}_{0.95}$ indicates more design freedom even under adverse parameter realizations. The second is the contraction ratio $\Gamma = (\mathcal {A}_{0.50} - \mathcal {A}_{0.95}) / \mathcal {A}_{0.50}$, which measures how much design space is lost when moving from median to high-confidence feasibility; values near unity indicate brittle architectures whose median performance does not translate to reliable feasibility. The third is the maximum total-order Sobol index $\max _i S_{T_i}$, which penalizes architectures whose feasibility depends heavily on a single uncertain parameter. The ARI is then:

12 $$\begin{aligned} ARI = \mathcal {A}_{0.95} \cdot (1 - \Gamma ) \cdot (1 - \max _i S_{T_i}) \end{aligned}$$

Design point selection follows a two-stage protocol formalized in Algorithm 2. In Phase 1, the architecture with the highest ARI is selected. In Phase 2, within that architecture’s 95%-confidence feasible region, the design point is placed at a weighted blend of the robustness optimum (maximizing distance from all constraint boundaries) and the feasible centroid, with weight *λ = 0.3* toward the optimum (biased toward the centroid for safety-critical applications). Margin verification confirms that the probability of satisfying all constraints simultaneously exceeds 98% at the selected point.

**Algorithm 2:**
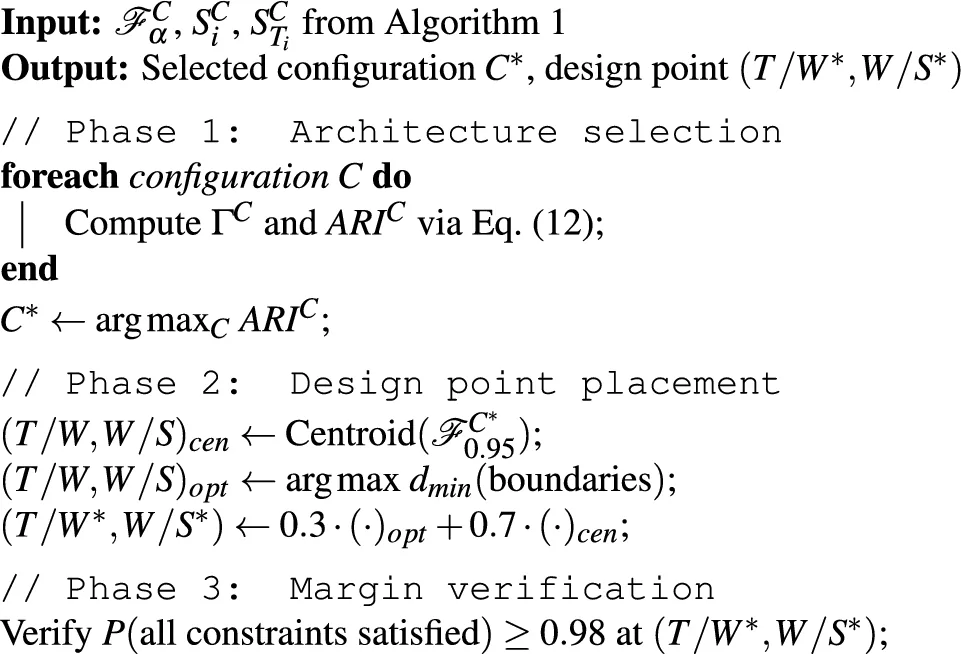
Architecture comparison and design point selection.

### MTOW closure and geometry derivation

At the selected design point, iterative weight closure converts probabilistic sizing parameters into physical vehicle geometry for each Monte Carlo sample—the concrete aircraft dimensions that a medical delivery program would need to specify for procurement, airworthiness certification, and operational infrastructure planning. The iteration starts from an initial MTOW estimate and updates by computing the total mission energy (hover, cruise, climb, auxiliary, and reserve), deriving the required battery mass fraction $f_{batt} = E_{total} / (e_{batt} \cdot W_{MTO})$, and solving for $W_{MTO} = W_{pay,eff} / (1 - f_{we,eff} - f_{batt})$. The MTOW-coupled parachute penalty ($\tilde{c} \cdot W_{MTO}$) creates a positive feedback loop: increasing MTOW increases parachute mass, which further increases MTOW. Convergence is guaranteed because the loop gain $\tilde{c} \in [0.04, 0.08]$ is well below unity—each kilogram of MTOW increase adds at most 80 g of parachute mass, producing a rapidly converging geometric series. The iteration uses 50% relaxation for stability and converges in 3–5 steps to a relative tolerance of 0.5%. When the iteration produces $W_{MTO}> 25$ kg (the regulatory limit), the sample is classified as infeasible. Each converged sample yields the wing reference area $S = W_{MTO}\,g / (W/S)^*$, wing span $b = \sqrt{AR \cdot S}$, mean chord *c = S/b*, and rotor diameter $d_r = 2\sqrt{W_{MTO}\,g/(N_r\,\pi \,DL)}$. The ensemble of converged samples produces probability distributions for all geometric parameters, enabling visualization of the family of physical aircraft that satisfies the sizing constraints under the full uncertainty specification.

## Supplementary Information

Supplementary Information 1.

## Data Availability

The Python source code implementing the probabilistic constraint analysis framework and generating all figures and tables in this paper is available from the corresponding author upon reasonable request.

## Roman symbols

*AR*: Wing aspect ratio [–]
*b*: Wing span [m]
*c*: Wing mean chord [m]
$$C_D$$: Drag coefficient [–]
$C_{D_0}$: Zero-lift drag coefficient [–]
$C_{D,sr}$: Stopped-rotor drag coefficient (L+C only) [–]
$$C_L$$: Lift coefficient [–]
$C_{L_{max}}$: Maximum lift coefficient [–]
$$d_r$$: Rotor diameter [m]
*DL*: Disc loading [N/m$^2$]
*e*: Oswald span efficiency factor [–]
$e_{batt}$: Battery pack specific energy [Wh/kg]
*E*: Energy [J or Wh]
$f_{batt}$: Battery mass fraction [–]
$f_{dl}$: Hover download fraction (T-R only) [–]
$f_{res}$: Energy reserve fraction [–]
$f_{we}$: Empty weight fraction [–]
*h*: Altitude above mean sea level [m]
*K*: Induced drag factor, $K = 1/(\pi \,e\,AR)$ [–]
$k_{red}$: Propulsion redundancy factor [–]
*L*: ISA temperature lapse rate [K/m]
*L*/*D*: Lift-to-drag ratio [–]
$N_r$: Number of lift rotors [–]
*P*: Power [W]
$P_{aux}$: Total auxiliary power draw [W]
*R*: Mission range (one way) [km]
*RoC*: Rate of climb [m/s]
*S*: Wing reference area [m$^2$]
$S_i$: First-order Sobol index of parameter *i* [–]
$S_{T_i}$: Total-order Sobol index of parameter *i* [–]
*T*: Thrust [N]
*T*/*W*: Thrust-to-weight ratio [–]
$$T_{amb}$$: Ambient temperature [$$^\circ {C}$$]
$v_i$: Rotor induced velocity [m/s]
$$V_{cr}$$: Cruise speed [m/s]
$$V_{stall}$$: Stall speed [m/s]
$$V_w$$: Wind speed [m/s]
*W*/*S*: Wing loading [N/m$$^2$$]
$W_{MTO}$: Maximum take-off weight [kg]
$W_{pay}$: Payload mass (cargo) [kg]
$W_{pay,eff}$: Effective payload including RIK penalties [kg]

## Greek symbols

*α*: Confidence level [–]
*Γ*: Feasible area contraction ratio [–]
$\delta _{ctrl}$: Hover control margin [–]
*Δ h*: Terrain clearance altitude gain [m]
*Δ T*: Temperature deviation from ISA [$^\circ {C}$]
$\eta _{prop}$: Cruise propulsive efficiency [–]
$\eta _{prop,h}$: Hover propulsive efficiency (figure of merit) [–]
*ρ*: Air density [kg/m$^3$]
$$\sigma _\rho$$: Density ratio $$\rho /\rho _{SL}$$ [–]
*τ*: Rotor tilt angle (T-R only) [$^\circ$]

## Notation

$\tilde{x}$: Random variable (uncertain RIK coefficient)
$P_n$: *n*th percentile of a distribution
$\mathcal {N}(\mu , \sigma )$: Normal distribution with mean *μ* and std. dev. *σ*
$\mathcal {U}(a, b)$: Uniform distribution on [*a*, *b*]
$\text {Tri}(a, m, b)$: Triangular distribution: minimum *a*, mode *m*, maximum *b*
$\text {Wei}(\lambda , k)$: Weibull distribution: scale *λ*, shape *k*
CV: Coefficient of variation ($\sigma /\mu$)
$\mathcal {F}_\alpha$: Feasible region at confidence level *α*
$\mathcal {A}_\alpha$: Area of $\mathcal {F}_\alpha$ in the *T*/*W*–*W*/*S* plane
