# Supplementary material for: How stakeholder requirements constrain drone-based medical delivery: probabilistic sizing of eVTOL aircraft at high altitude
Source: Sci Rep. 2026 Jun 10;16:26444. doi: 10.1038/s41598-026-54743-2 (PMC13500619; doi:10.1038/s41598-026-54743-2)
Supplement: Supplementary file 1 [file 41598_2026_54743_MOESM1_ESM.pdf]

# Supplementary Information

## How Stakeholder Requirements Constrain Drone-Based Medical Delivery: Probabilistic Sizing of eVTOL Aircraft at High Altitude

Victor Alulema[1,2], Gloria Acosta-Vargas[3], Cesar Guevara[4], Patricia Acosta-Vargas[5,*]

[1]Department of Mechanical Engineering, LUAS-EPN, ATA Research Group, Escuela Politécnica Nacional, Quito, Ecuador
[2]EAVISE, PSI, Department of Electrical Engineering, KU Leuven, Belgium
[3]Medicine Faculty, Pontificia Universidad Católica del Ecuador, Quito, Ecuador
[4]CUNEF University, Quantitative Methods Department, Madrid, Spain
[5]Intelligent and Interactive Systems Laboratory, Universidad de Las Américas, Quito, Ecuador
[*]Corresponding author: patricia.acosta@udla.edu.ec

Supplementary Tables S1–S5 provide detailed numerical data supporting the Methods and Results sections. Supplementary Figures S1–S7 provide additional results visualizations. Supplementary Figures S8–S12 provide methodological diagrams and parameter distributions.

# Contents

**1 Supplementary Tables** 2

**2 Supplementary Figures from Results (S1–S7)** 4

**3 Supplementary Figures from Methods (S8–S12)** 8

**4 Additional Supplementary Items (Revision)** 11

# 1 Supplementary Tables

Supplementary Table 1: Key constraint analysis results at $h = 2{,}800$ m MSL. Referenced in Results.

| | **Standard ($V_{cr} = 20$ m/s)** | | **Urgent ($V_{cr} = 28$ m/s)** | |
|---|---|---|---|---|
| **Metric** | L+C | T-R | L+C | T-R |
| Hover $T/W$ (P50) | 2.92 | 3.33 | 2.92 | 3.33 |
| Hover $T/W$ (P5–P95) | 2.49–3.37 | 2.83–3.85 | 2.49–3.37 | 2.83–3.85 |
| Stall $W/S$ (P50) [N/m$^2$] | 88 | 88 | 88 | 88 |
| Energy min $W/S$ (P50) [N/m$^2$] | 35 | 38 | 60 | 63 |
| $W/S$ band width (P50) [N/m$^2$] | 53 | 50 | 28 | 25 |
| Sample feasibility [%] | 53 | 35 | 27 | 15 |

Supplementary Table 2: Requirement Impact Kit definitions. Referenced in Methods.

| **RIK** | **Mass model** | **Power model** | **Constraint** |
|---|---|---|---|
| Baseline (VLOS) | $\tilde{a} \sim \mathcal{N}(0.15,\ \text{CV}{=}0.15)$ kg | Tri$(3, 5, 8)$ W | — |
| CC-Passive | $0.5 + \tilde{b}\,W_{pay}$ kg, $\tilde{b} \sim \mathcal{U}(0.5,\ 1.1)$ | 0 | — |
| CC-Active | $\tilde{a} + \tilde{b}\,W_{pay}$ kg, $\tilde{a} \sim \mathcal{N}(0.4,\ \text{CV}{=}0.15)$, $\tilde{b} \sim \mathcal{U}(0.2,\ 0.45)$ | Tri$(15, 22, 30)$ W | — |
| BVLOS Avionics | $\tilde{a} \sim \mathcal{U}(0.35,\ 0.70)$ kg | Tri$(10, 18, 28)$ W | — |
| Safety (Para.+FTS) | $\tilde{a} + \tilde{c}\,W_{MTO}$ kg, $\tilde{a} \sim \mathcal{N}(0.15,\ \text{CV}{=}0.20)$, $\tilde{c} \sim \mathcal{U}(0.04,\ 0.08)$ | 0 | — |
| Redundancy (Quad) | — | — | $k_{red} = 4/3$ |
| Fast Delivery | — | — | $V_{cr} = 28$ m/s |

Supplementary Table 3: Requirement scenarios as progressive RIK combinations. Referenced in Methods.

| **Scenario** | **Active RIKs** | $V_{cr}$ |
|---|---|---|
| S0: Bare Cargo | Baseline | 20 m/s |
| S1: Basic Medical | Baseline + CC-Passive | 20 m/s |
| S2: Standard Medical | CC-Active + BVLOS + Safety + Redundancy | 20 m/s |
| S3: Urgent Medical | S2 + Fast Delivery | 28 m/s |
| S4: Extreme | S3 with Hex Redundancy ($k_{red} = 6/5$) | 28 m/s |

Supplementary Table 4: Uncertain parameter definitions. Type: A = aleatory, E = epistemic. Referenced in Methods.

| **Param.** | **Unit** | **Distribution** | **Type** | **Rationale** |
|---|---|---|---|---|
| $W_{pay}$ | kg | $\mathcal{U}(0.5, 2.0)$ | A | Blood bag (0.45 kg) to multi-unit (2 kg) |
| $R$ | km | $\mathcal{N}(40, 10)$ | A | Health facility network distances |
| $T_{amb}$ | °C | $\mathcal{N}(15, 7)$ | A | Quito annual climate statistics |
| $V_w$ | m/s | Wei($\lambda=5$, $k=2$) | A | Andean wind Weibull fit |
| $C_{D_0}$ | — | $\mathcal{N}(0.035$, CV $= 0.15)$ | E | Wetted area estimation scatter |
| $e$ | — | $\mathcal{N}(0.75, 0.05)$ | E | Empirical range for AR = 8 |
| $C_{L_{max}}$ | — | Tri(1.2, 1.4, 1.6) | E | Clean to slotted flap range |
| $\eta_{prop}$ | — | Tri(0.65, 0.75, 0.80) | E | Small propeller test data range |
| $\eta_{prop,h}$ | — | Tri(0.45, 0.55, 0.65) | E | Rotor figure of merit range |
| $e_{batt}$ | Wh/kg | $\mathcal{N}(180, 20)$ | E | Pack-level mid-life performance |
| $\delta_{ctrl}$ | — | $\mathcal{U}(0.25, 0.45)$ | E | Calm to gusty hover margin |
| $C_{D,sr}$ | — | $\mathcal{U}(0.3, 0.8)$ | E | Blade alignment sensitivity |
| $f_{dl}$ | — | $\mathcal{U}(0.10, 0.15)$ | E | T-R download fraction range |
| $P_{cool}$ | W | Tri(15, 22, 30) | E | Thermal system design range |
| $P_{av}$ | W | Tri(10, 18, 28) | E | BVLOS avionics suite range |
| $f_{we}$ | — | $\mathcal{N}(0.45$, CV$=0.08)$ | E | Composite small UAV statistics |

Supplementary Table 5: Fixed design parameters. Referenced in Methods.

| **Parameter** | **Symbol** | **Value** | **Source / Justification** |
|---|---|---|---|
| Stall speed | $V_{stall}$ | 12 m/s | Small UAV certification practice |
| Rate of climb | $RoC$ | 4 m/s | Operational requirement |
| Wing aspect ratio | $AR$ | 8 | High-efficiency small UAV range |
| Hover time | $t_{hover}$ | 180 s | TO + landing + loiter margin |
| Number of lift rotors | $N_r$ | 4 | Quad configuration baseline |
| Disc loading | $DL$ | 120 N/m$^2$ | Small eVTOL rotor practice |
| MTOW limit | $W_{MTO,max}$ | 25 kg | Regulatory boundary |
| Energy reserve fraction | $f_{res}$ | 0.10 | Standard 10% reserve |
| Altitude gain | $\Delta h$ | 300 m | Typical Quito terrain clearance |
| Climb efficiency factor | $\eta_{climb}$ | 0.85 | Drag overhead during climb |

## 2 Supplementary Figures from Results (S1–S7)

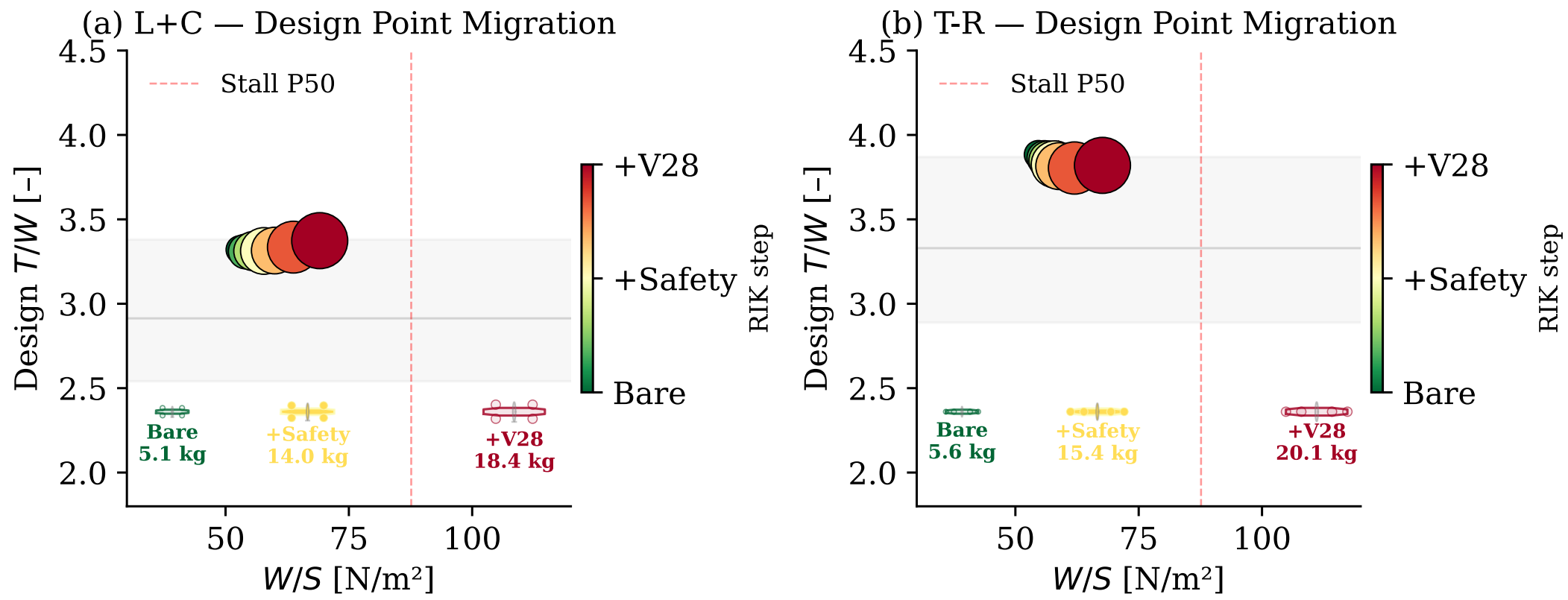


Supplementary Figure 1: Design point migration as medical delivery requirements accumulate. (a) L+C, (b) T-R. Marker size ∝ MTOW; color from green (bare) to red (full urgent). Inset silhouettes show the physical aircraft at selected requirement levels.

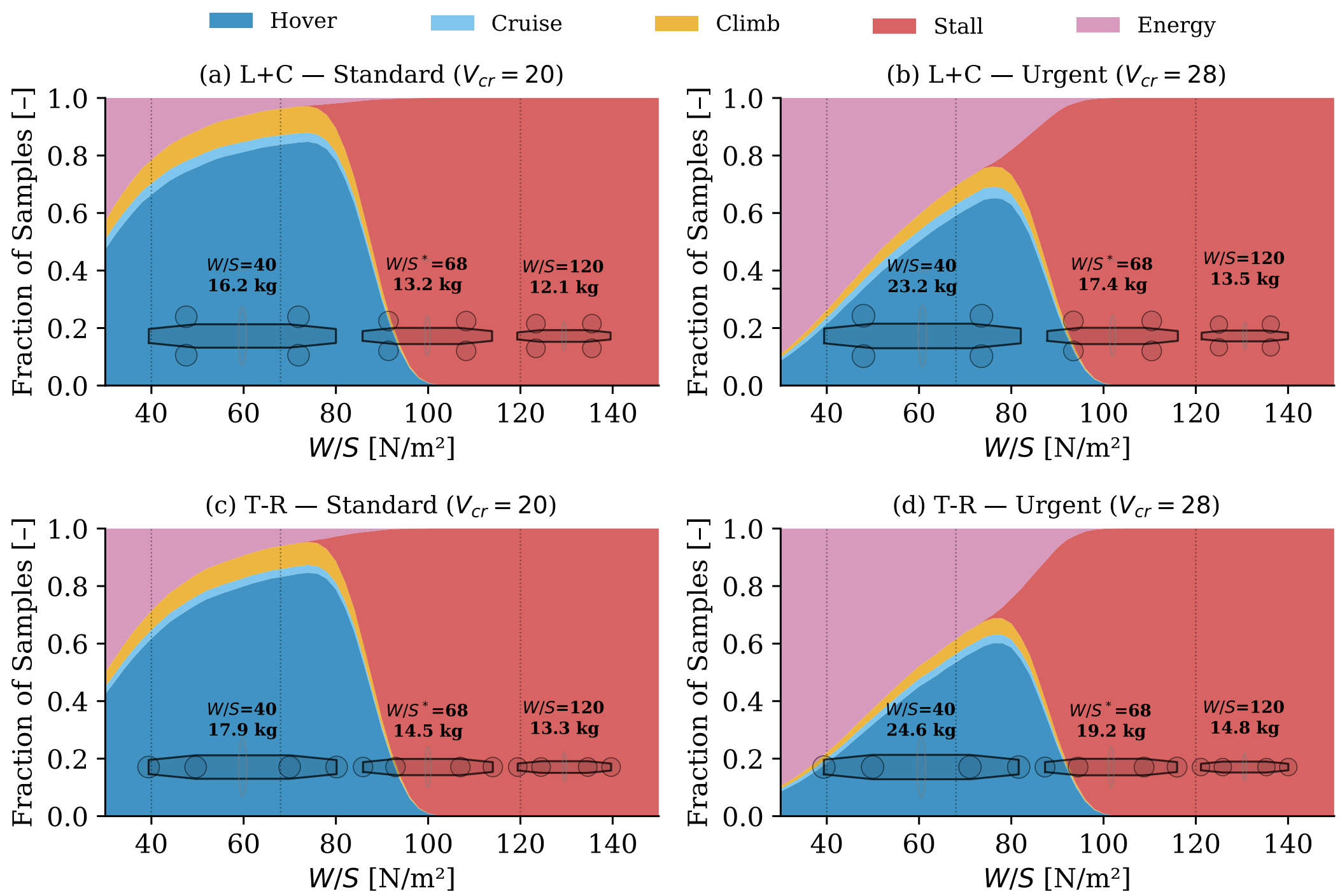


Supplementary Figure 2: Constraint competition map showing the proportional $T/W$ contribution of each constraint at each $W/S$. Top row: L+C. Bottom row: T-R. Left: Standard. Right: Urgent.

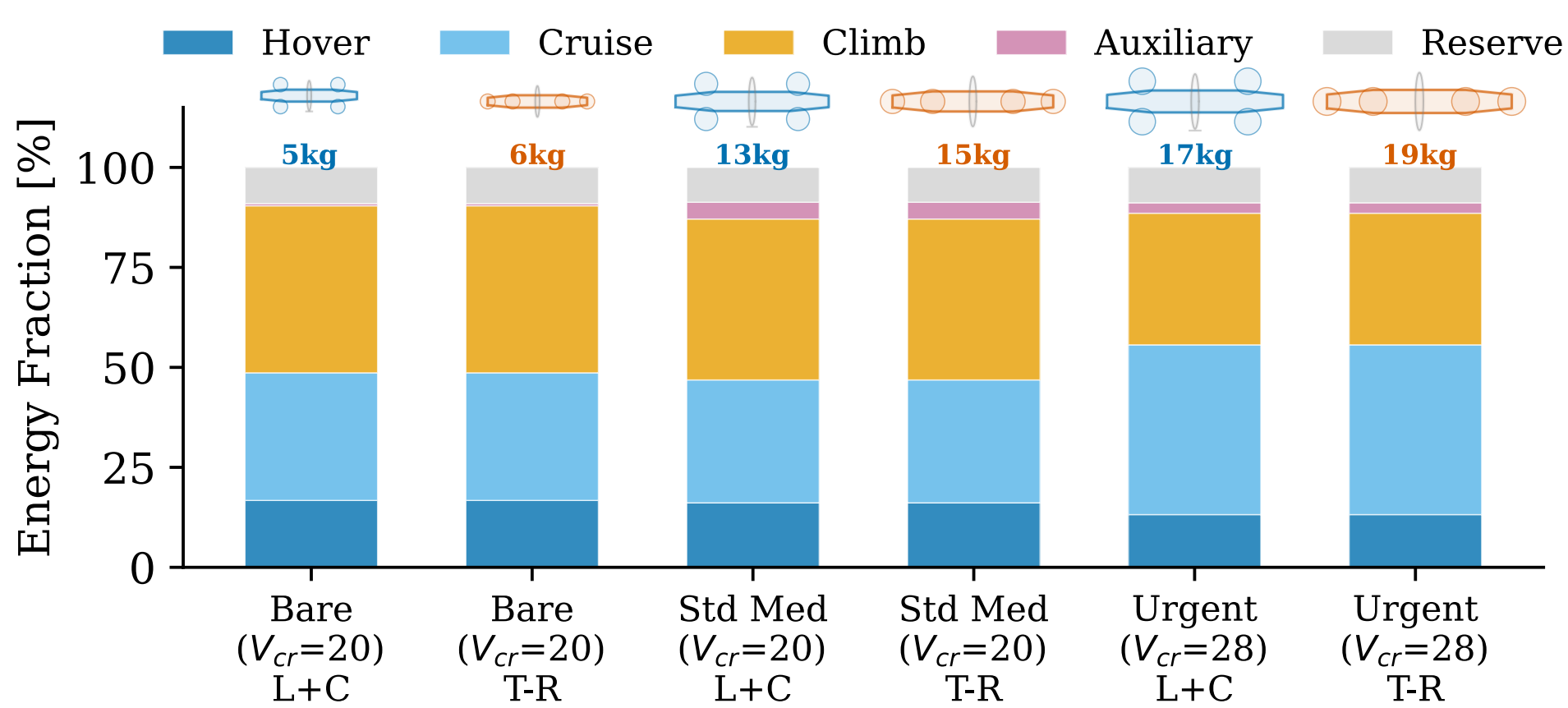


Supplementary Figure 3: Mission energy budget breakdown by scenario and configuration at the design point.

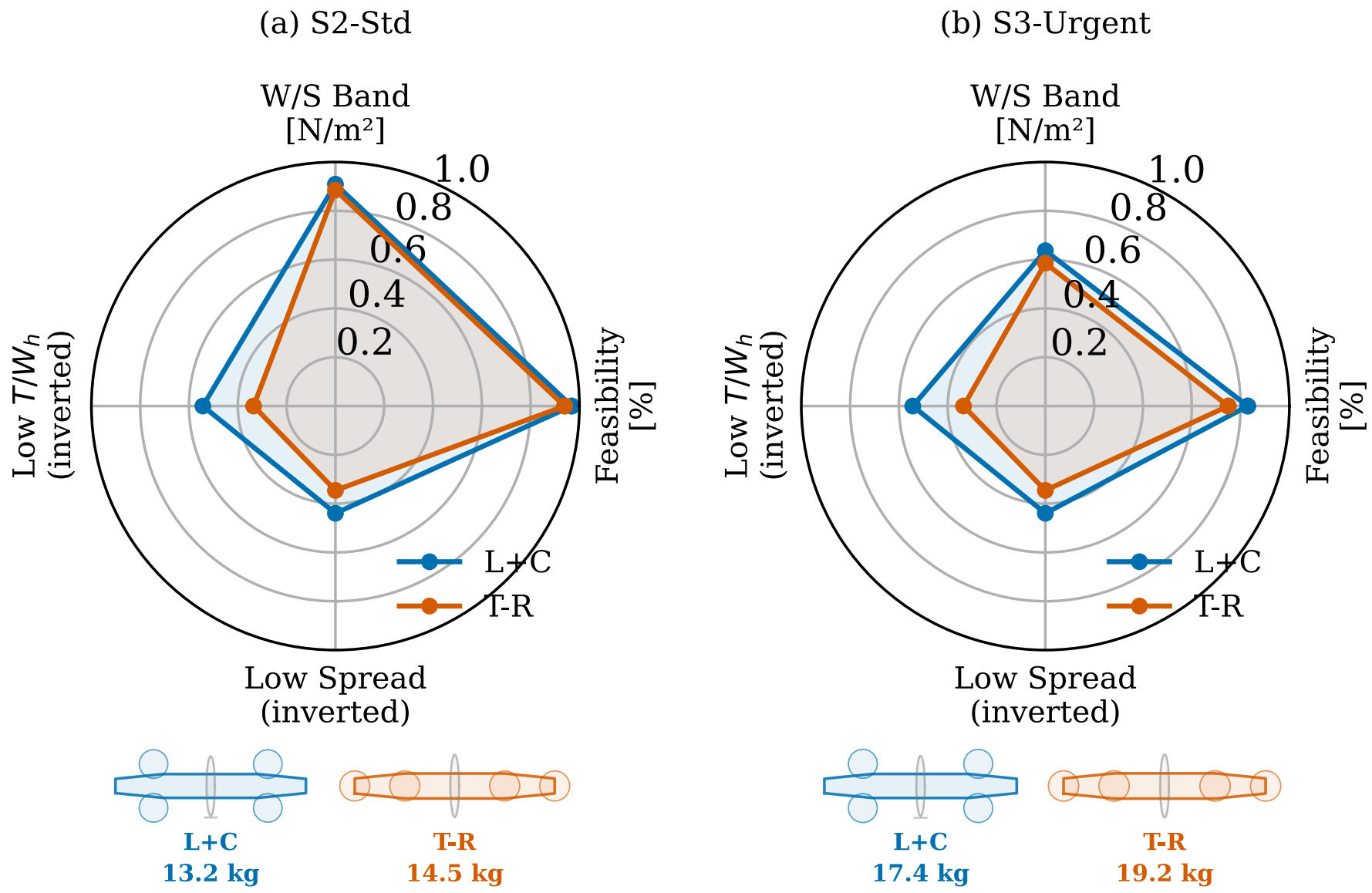


Supplementary Figure 4: Architecture Robustness Index decomposition. (a) Standard Medical, (b) Urgent Medical.

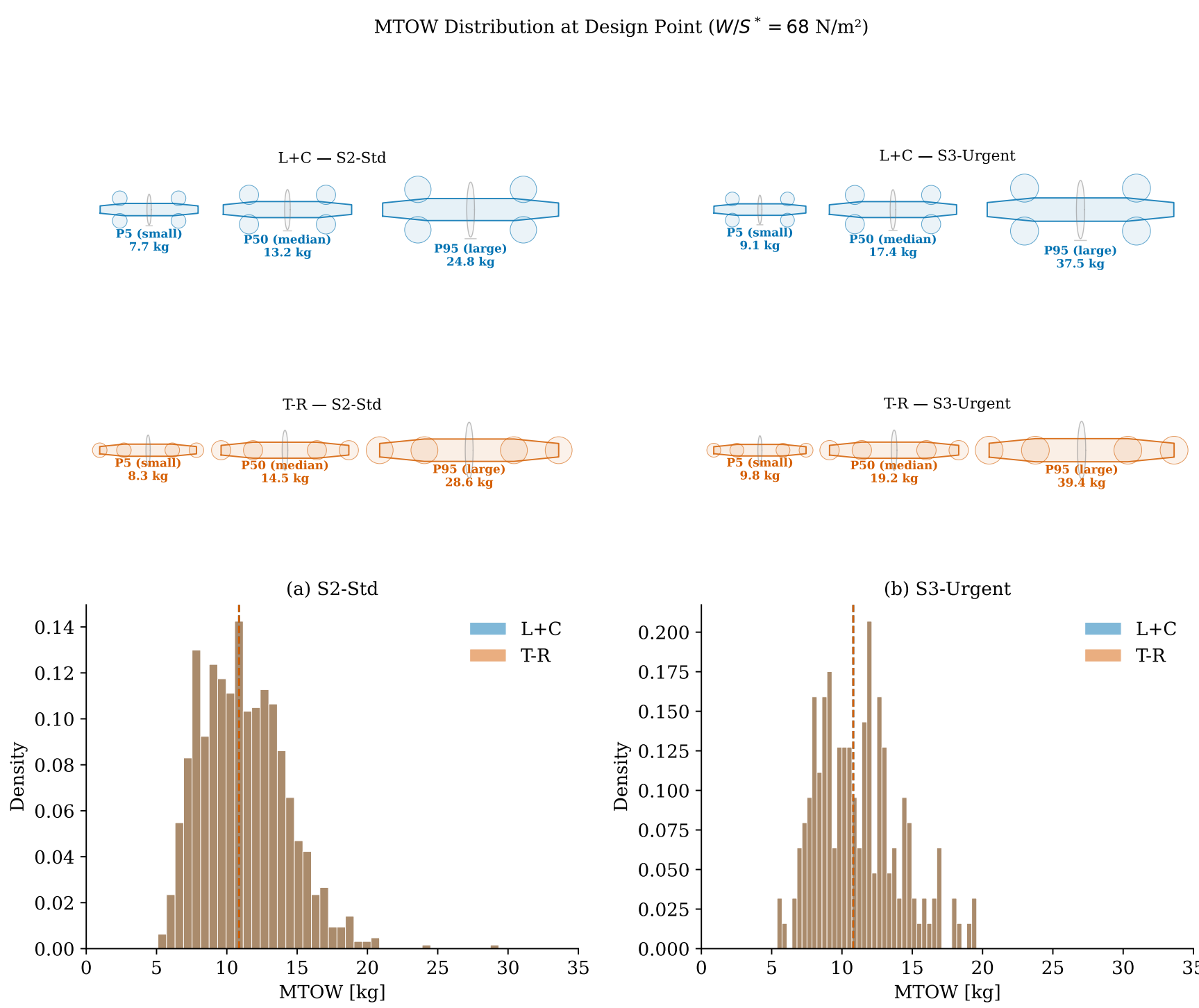


Supplementary Figure 5: MTOW probability distributions at $W/S^* = 68$ N/m$^2$ for the Standard Medical scenario.

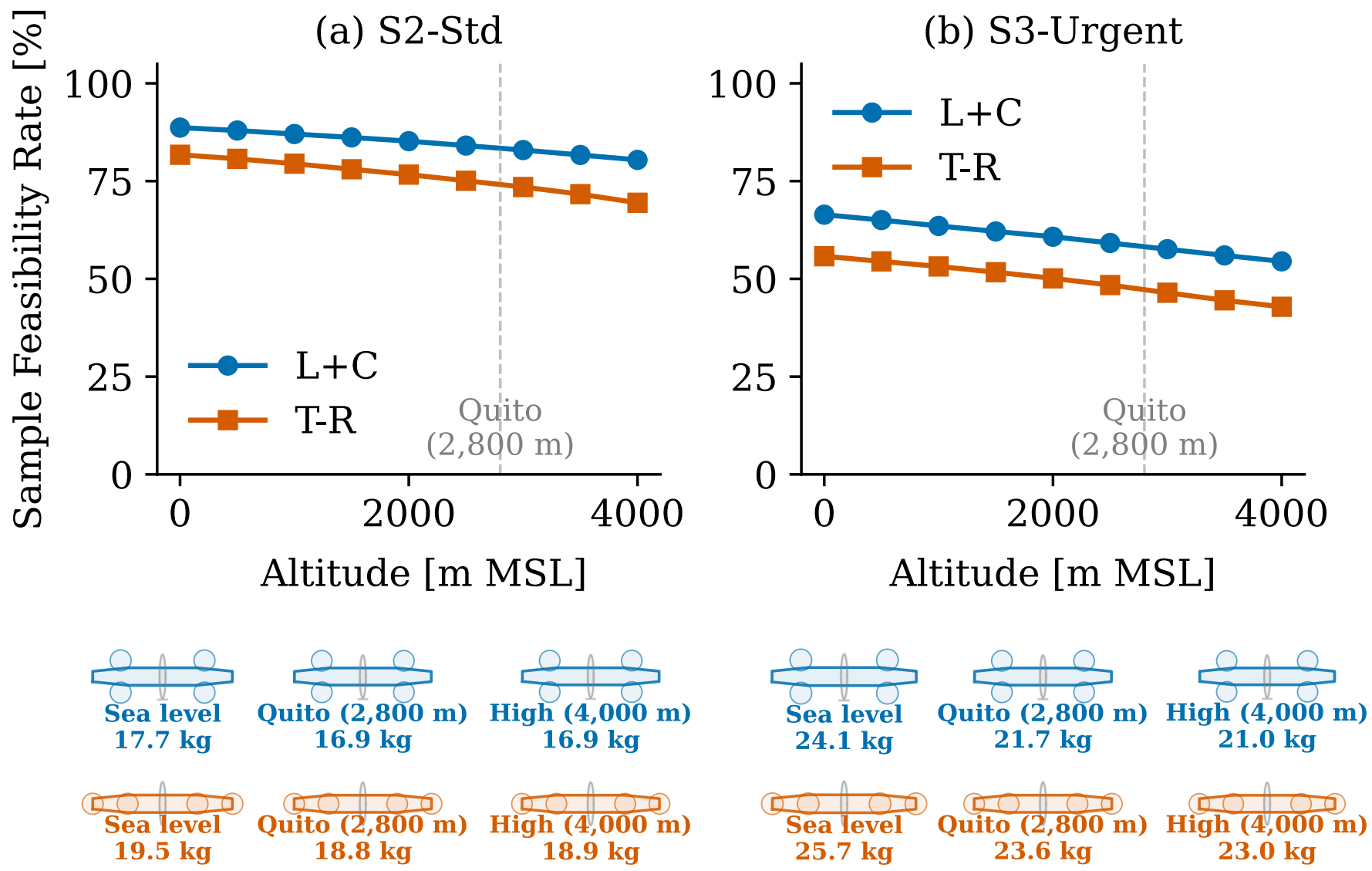


Supplementary Figure 6: Feasibility degradation with altitude for Standard and Urgent Medical scenarios.

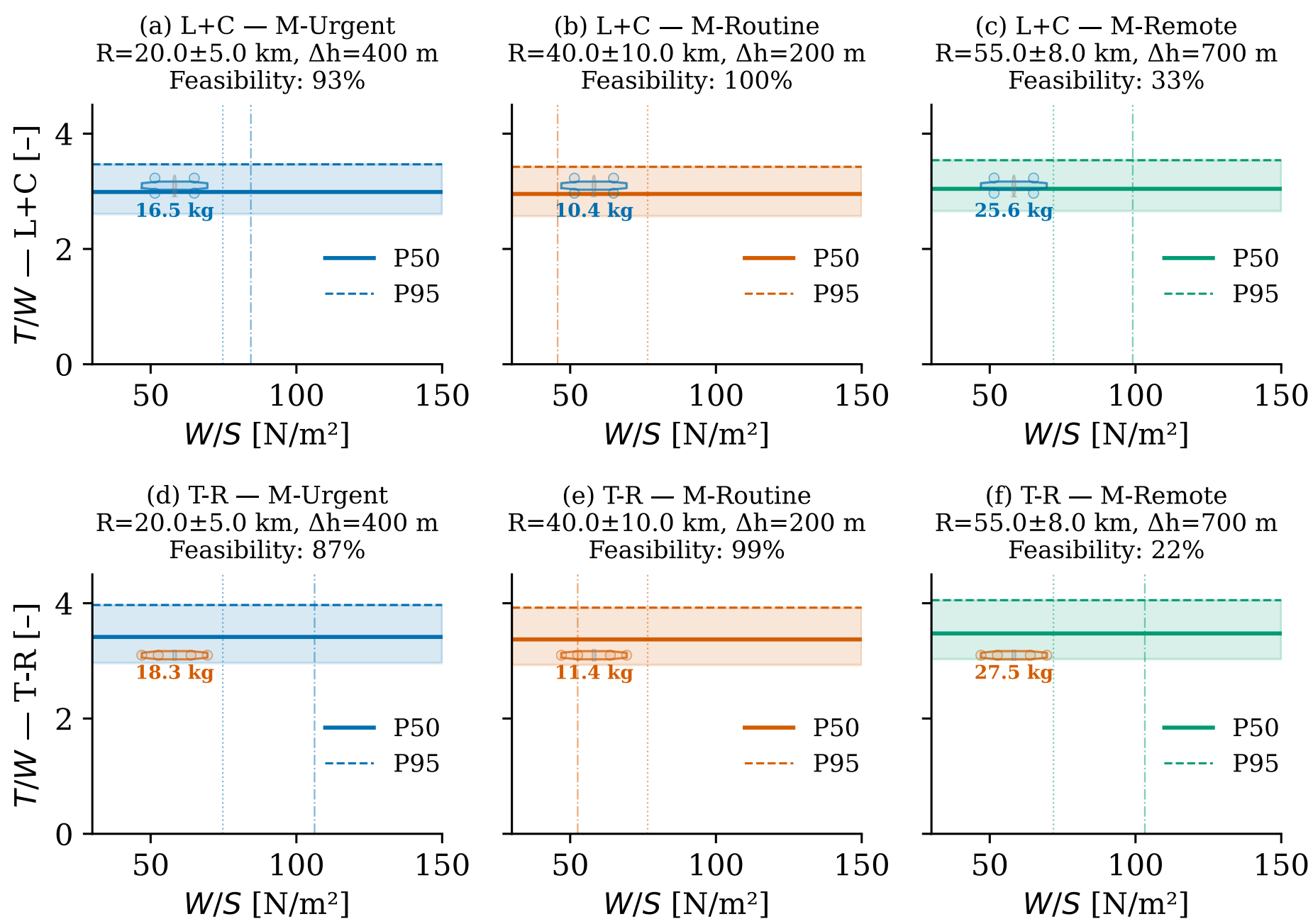


Supplementary Figure 7: Multi-mission profile comparison for L+C. Routine: 95%. Urgent: 67%. Remote: 8%.

# 3 Supplementary Figures from Methods (S8–S12)

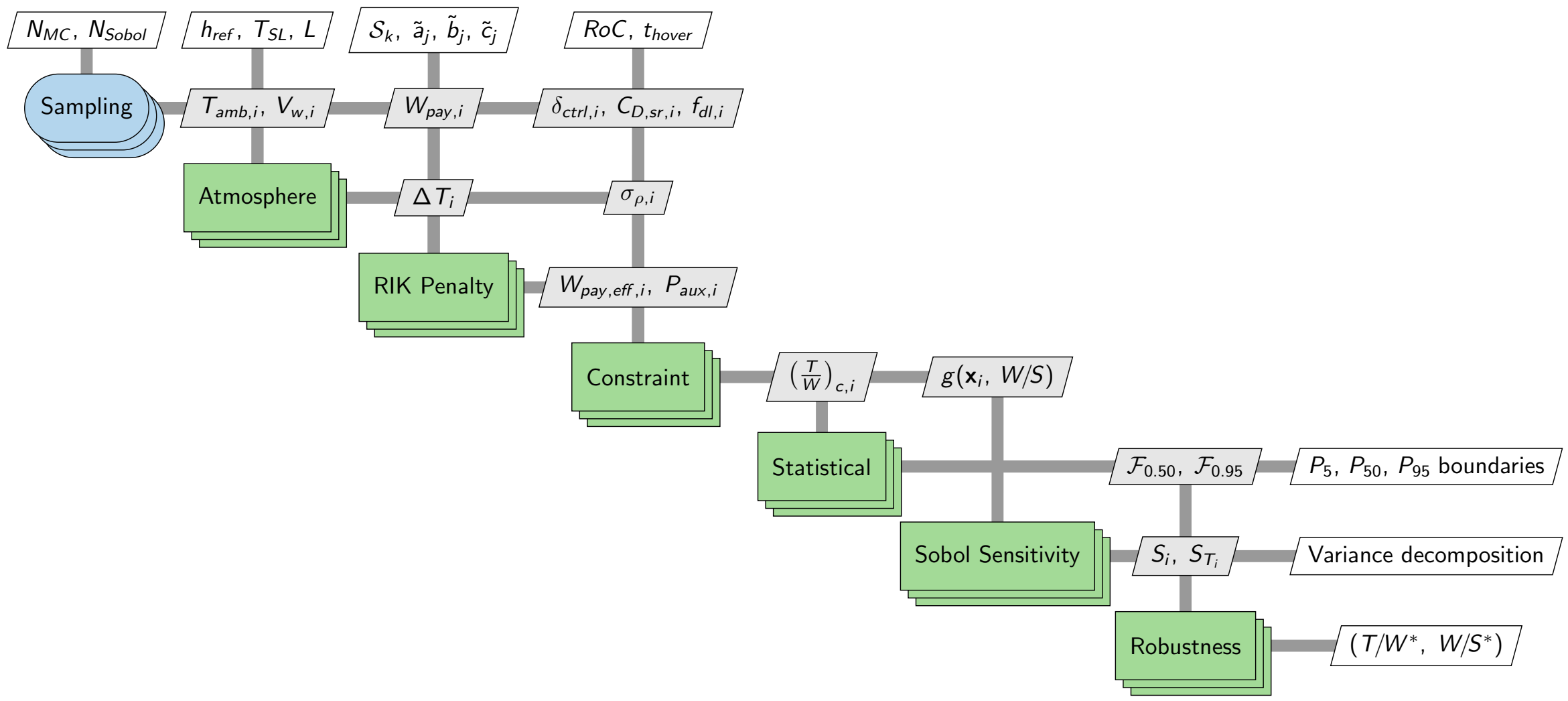


Supplementary Figure 8: XDSM of the probabilistic constraint analysis framework.

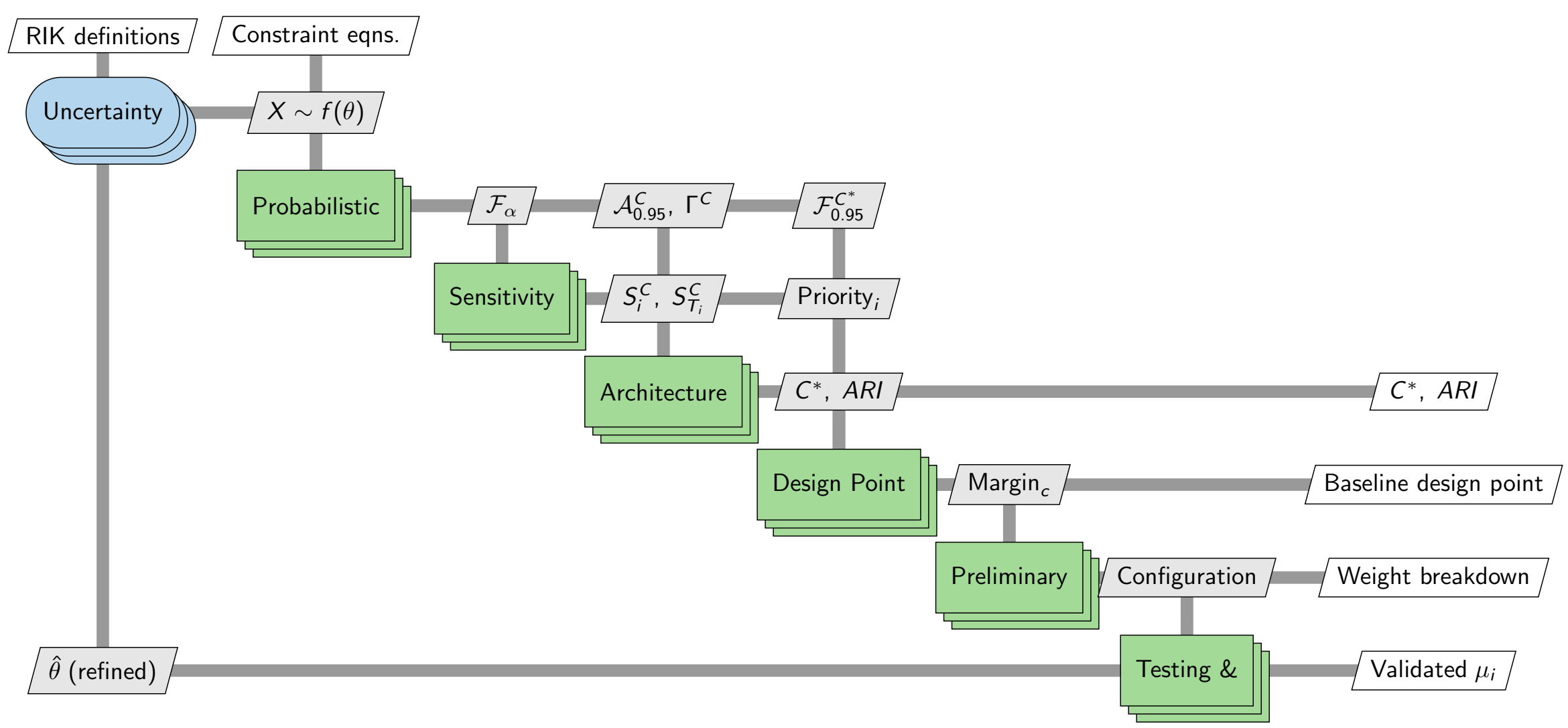


Supplementary Figure 9: XDSM of the iterative design refinement protocol.

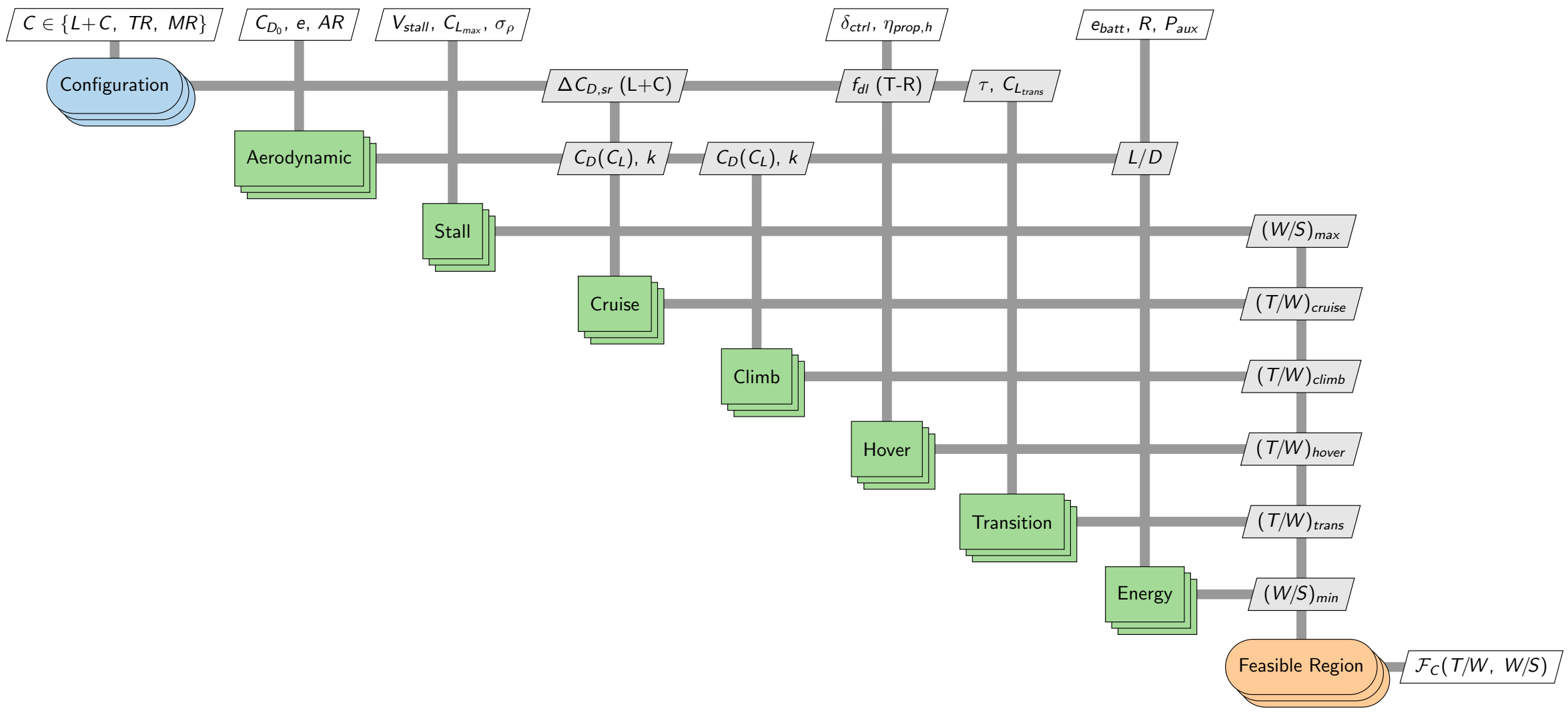


Supplementary Figure 10: XDSM of the constraint evaluation module.

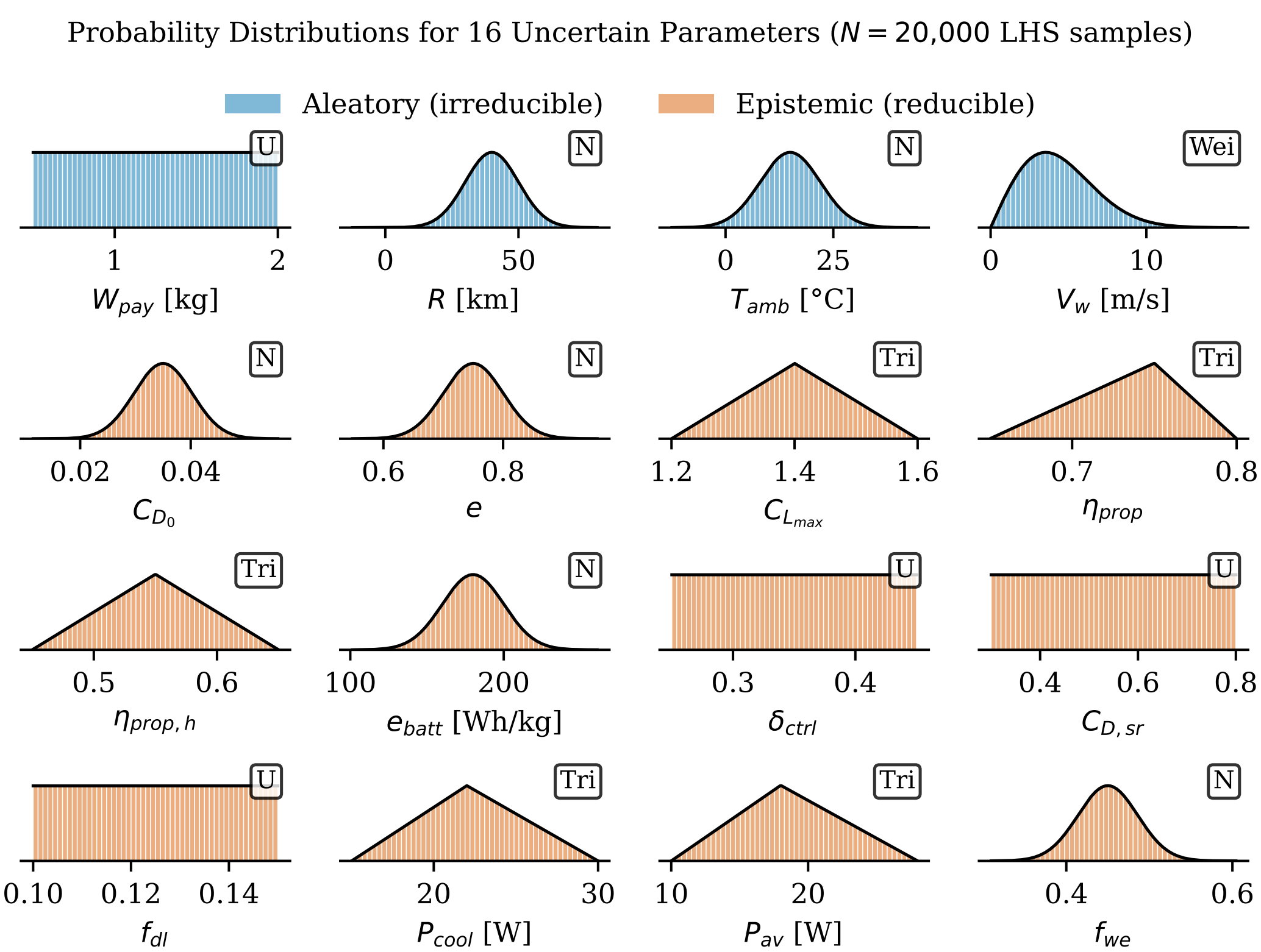


Supplementary Figure 11: Probability distributions for the 16 uncertain parameters ($N = 20{,}000$ LHS samples). Blue: aleatory. Orange: epistemic. Black curves: theoretical PDFs.

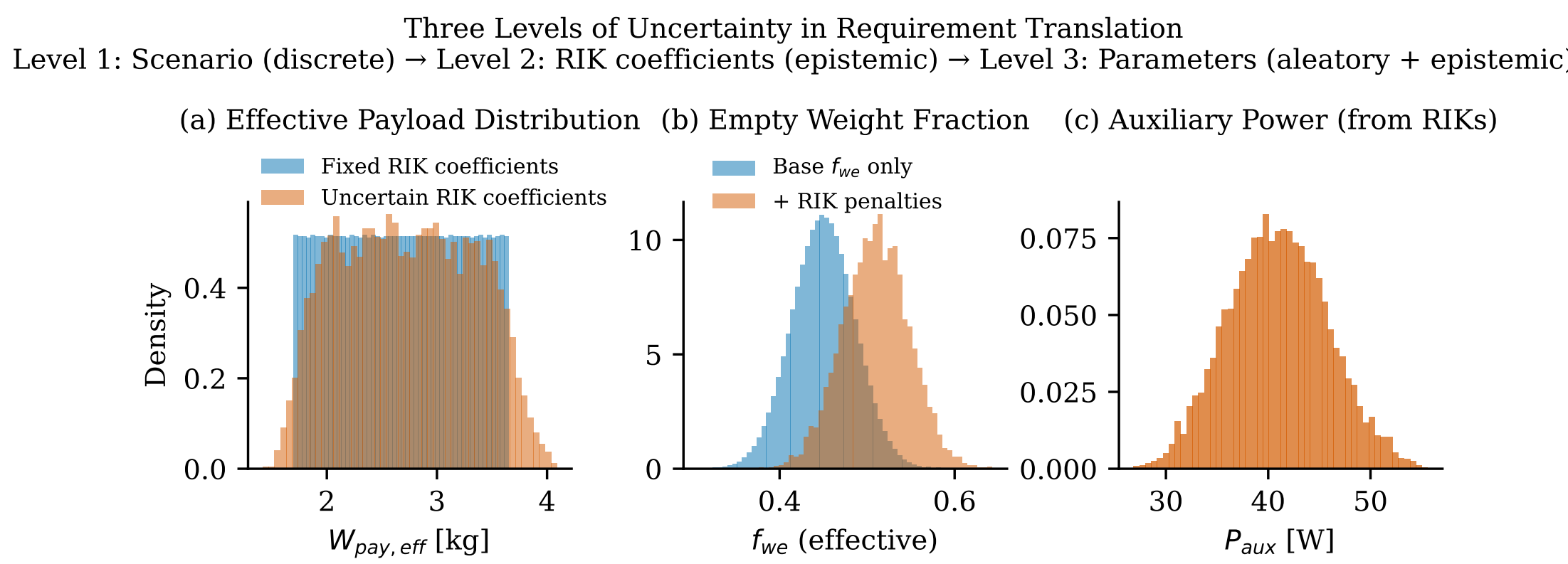


Supplementary Figure 12: Three-level uncertainty compounding for the Standard Medical scenario (S2). (a) Effective payload. (b) Effective empty weight fraction. (c) Auxiliary power.

# 4 Additional Supplementary Items (Revision)

Supplementary Table 6: Comparison of feasibility metrics under three analysis approaches for L+C Standard Medical at $h$ = 2,800 m MSL. The deterministic approach masks the 86% reduction in feasible $W/S$ band width that emerges under probabilistic analysis at 95% confidence.

| **Approach** | **Band feas. [%]** | **Band$_{50}$ [N/m$^2$]** | **Band$_{95}$ [N/m$^2$]** |
|---|---|---|---|
| Deterministic (nominal) | 100 (apparent) | 57.6 | 57.6 |
| Single-level UQ (Level-3 only) | 97.5 | 56.1 | 9.4 |
| Three-level RIK framework | 96.8 | 57.7 | 7.8 |

Supplementary Table 7: ARI sensitivity to index formulation for L+C versus T-R. The L+C ranks first under the multiplicative, geometric mean, and minimax formulations for the Standard scenario. All formulations show $A_{95}$ = 0 for both architectures under Urgent conditions, indicating that the 95%-confidence feasible region vanishes and requirement relaxation is necessary.

| **Scenario** | **Config** | $\mathcal{A}_{95}$ | $1-\Gamma$ | $1-\max S_T$ | **ARI (mult.)** | **ARI (w. sum)** |
|---|---|---|---|---|---|---|
| Standard | L+C | 0.065 | 0.135 | 0.260 | 0.0023 | 0.145 |
| Standard | T-R | 0.000 | 0.000 | 0.281 | 0.0000 | 0.084 |
| Urgent | L+C | 0.000 | 0.000 | 0.260 | 0.0000 | 0.078 |
| Urgent | T-R | 0.000 | 0.000 | 0.281 | 0.0000 | 0.084 |

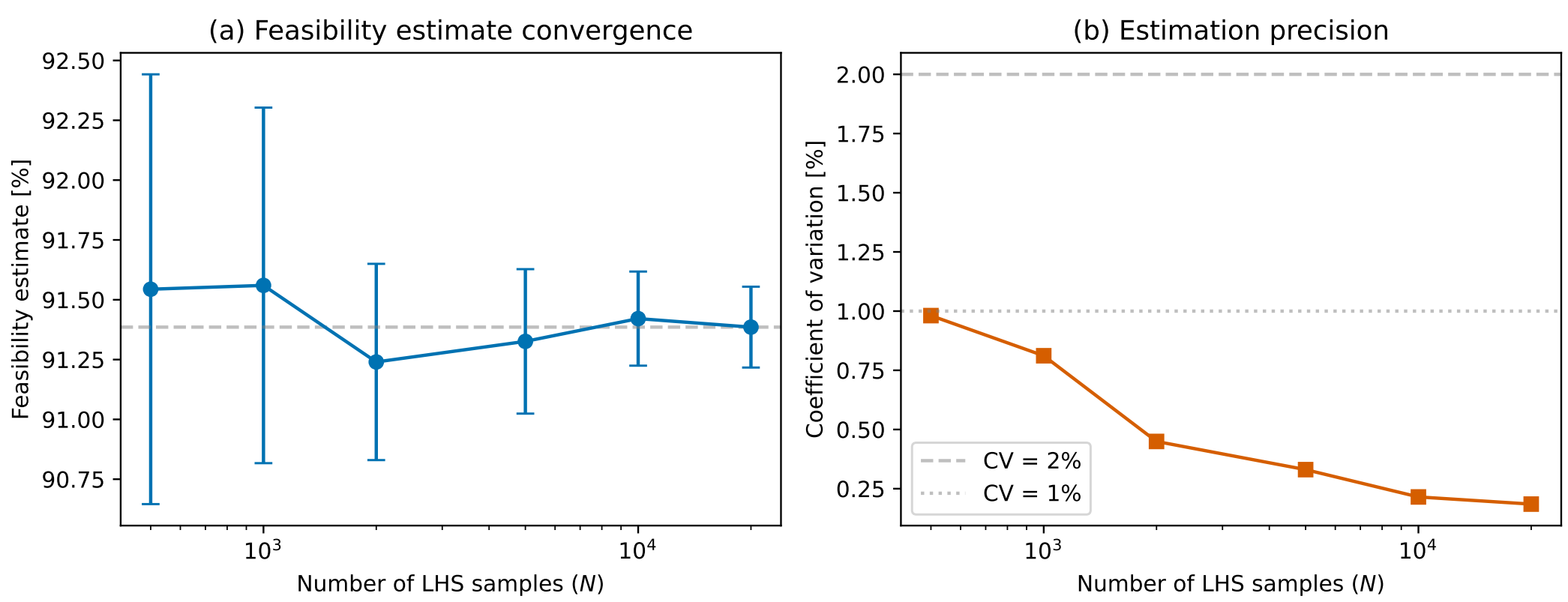


Supplementary Figure 13: Convergence of feasibility estimates with increasing LHS sample size for L+C Standard Medical scenario ($h$ = 2,800 m MSL). (a) Feasibility estimate stabilizes by $N$ = 5,000. (b) Coefficient of variation (from 50 bootstrap replicates) drops below 1% at $N$ = 5,000 and to 0.2% at $N$ = 10,000.
